# Magnetic resonance control of reaction yields through genetically-encoded protein:flavin spin-correlated radicals in a live animal

**DOI:** 10.1101/2025.02.27.640669

**Published:** 2025-03-03

**Authors:** Shaun C. Burd, Nahal Bagheri, Maria Ingaramo, Alec F. Condon, Samsuzzoha Mondal, Dara P. Dowlatshahi, Jacob A. Summers, Srijit Mukherjee, Andrew G. York, Soichi Wakatsuki, Steven G. Boxer, Mark Kasevich

**Affiliations:** 1 Department of Physics, Stanford University, Palo Alto, 94305, USA.; 2 Department of Chemistry, Stanford University, Palo Alto, 94305, USA.; 3 Department of Electrical Engineering, Stanford University, Palo Alto, 94305, USA.; 4 Calico Life Sciences, South San Francisco, 94080, USA.; 5 Department of Biology, Stanford University, Palo Alto, 94305, USA.; 6 Department of Structural Biology, Stanford University, Palo Alto, 94304, USA.; 7 SLAC National Accelerator Laboratory, Menlo Park, 94025, USA.

## Abstract

Radio-frequency (RF) magnetic fields can influence reactions involving spin-correlated radical pairs. This provides a mechanism by which RF fields can influence living systems at the biomolecular level. Here we report the modification of the emission of various red fluorescent proteins (RFPs), in the presence of a flavin cofactor, induced by a combination of static and RF magnetic fields. Resonance features in the protein fluorescence intensity were observed near the electron spin resonance frequency at the corresponding static magnetic field strength. This effect was measured at room temperature both in vitro and in the nematode *C. elegans*, genetically modified to express the RFP mScarlet. These observations suggest that the magnetic field effects measured in RFP-flavin systems are due to quantum-correlated radical pairs. Our experiments demonstrate that RF magnetic fields can influence dynamics of reactions involving RFPs in biologically relevant conditions, and even within a living animal. These results have implications for the development of a new class of genetic tools based on RF manipulation of genetically-encoded quantum systems.

Remote regulation of biomolecular reactions in living organisms could enable new scientific advances and potential therapies. Radio-frequency (RF) magnetic fields are ideal for this purpose as biological tissues are essentially transparent to this part of the electromagnetic spectrum. Here we demonstrate control of reaction yields using RF magnetic fields in a protein system genetically encoded in a living animal.

Although energy scales associated with typical static and RF magnetic fields are orders of magnitude below thermal fluctuations in biologically relevant conditions, RF fields can alter reaction yields in chemical and biochemical systems involving spin-correlated radical pairs (SCRPs) - a phenomenon known as reaction-yield detected magnetic resonance (RYDMR) (1–3). This is observed when a time-varying magnetic field is applied near the electron spin resonance (ESR) frequency $f_{ESR}=\;g\mu_{0}B_{0}/h$ in a static magnetic field $B_{0}$, where g is the electron g factor, $\mu_{0}$ is the Bohr magneton, and h is Planck’s constant. Moreover, observations that RF fields can influence biological processes in animals, including the magnetic sense of birds (4), have also been attributed to radical-pair dynamics (5). However, the detailed mechanism behind these effects at the protein level remains uncertain (6). Previous studies of radical pairs in protein systems (7–14) have focused on the effects of static magnetic field effects (MFEs), with the notable exception of chemically-modified photosynthetic reaction centers, where the influence of time-varying magnetic fields has been investigated extensively in vitro (15–17).

Here, we show that RF fields applied near the ESR frequency can modulate reaction yields in a system consisting of a red fluorescent protein (RFP) paired with a photoproduct of Flavin mononucleotide (FMN) (Fig. 1). Reaction yields in this system are inferred from changes in the RFP fluorescence intensity induced by the RF field. RFPs have become essential tools in biological and photophysical research (18, 19) and FMN is a naturally occurring molecule in cells. We present in vitro results showing RYDMR for various RFPs including mScarlet, mScarlet-I (20), mCherry (21), and mCherry-XL (22) together with FMN, and in vivo measurements in an animal, a transgenic *C. elegans* nematode, engineered to express mScarlet in all cells (23, 24).

A custom apparatus (Fig. S1) enables widefield fluorescence imaging of biological samples expressing FPs or purified proteins in aqueous solution inside an RF resonator. We typically use a bridged loop-gap resonator (BLGR) (25, 26) with a resonance frequency $f_{res}\sim 450\;MHz$ and a quality factor of ~ 600 (see SI). Static fields from 0 to ~ 30 mT parallel to $\left(B_{0ǁ}\right)$ or perpendicular to $\left(B_{0⟘}\right)$ the RF magnetic field $B_{1}$ can be generated using two sets of Helmholtz coils. For photoexcitation, lasers at 440 nm (blue) and 520 nm (green) are applied to the sample. We typically apply an initial ~10 s duration 440 nm photosensitization pulse that creates an as-yet not characterized photoproduct of the flavin, and subsequently apply 520 nm excitation for the remainder of the experiment (see SI). Fluorescence from the sample passes through a 650 nm long-pass filter and is imaged using a camera. The filter cuts off residual FMN fluorescence and serves to block scattered excitation light. Aqueous samples are placed inside a 3 mm inner diameter quartz tube, while *C. elegans* samples are placed on an agarose substrate on a fused-silica support, both near the center of the RF resonator.

Measurements of the fluorescence from an aqueous solution of purified mScarlet-I together with FMN pre-excited with blue light are shown in Fig. 2 A. Increasing the magnetic field $B_{0}$ initially results in a decrease in fluorescence, as observed in a recent report (27). Near $B_{0}=15.9\;mT$ — the field required for ESR at the RF frequency $f_{RF}=447\;MHz$ — there is a resonance feature in the fluorescence. Increasing the RF magnetic field amplitude $B_{1}$ results in a linear increase in the peak of the resonance and also an increase in the resonance full width at half maximum (FWHM) (Fig. 2 B and C) as estimated by using a least-squares fit of the sum of two Lorentzian functions to the data in Fig. 2 A (see SI). These features are consistent with RYDMR theory for the regime where $B_{1}$ is weak compared to the interactions driving spin-state mixing (3, 28). The fit is also used to estimate the MFE, quantified by the fractional reduction in the fluorescence intensity of the FP when the static magnetic field is applied, and $B_{1/2}$ — the field at which the fluorescence has decreased by half the estimated maximum possible value. The independence of both the estimated maximum MFE and $B_{1/2}$ values from the RF excitation strength $B_{1}$ suggests that the system is only weakly perturbed by the RF, as expected in the limit $B_{1}\ll B_{0}$ (Fig. 2 D and E). To measure the dynamic response of the resonance fluorescence to changes in $B_{1}$, the RF field was stepped between a given value of $B_{1}$ and zero within 500 *μ*s as shown in Fig. 2 F, with optical excitation as indicated in the figure. The dynamic response was inferred by fitting a first-order exponential decay model to the data (Fig. 2 G, see SI), to estimate the time constant (τ) of the response and the RYDMR amplitude (Figs. 2 H and I). We find τ to be largely independent of $B_{1}$ (Fig. 2 H), for a fixed value of the 520 nm excitation intensity $\left(I_{520}\right)$. Increasing $I_{520}$ from 0.5 W/cm^2^ to 3.8 W/cm^2^ results in a 2-fold increase in the magnetic resonance amplitude and a concomitant reduction in τ from ~ 2.5 s to ~ 1.5 s (Figs. 2H and I). As $I_{520}$ increases, the increase in the RYDMR amplitude becomes smaller, suggesting that the optical interaction becomes saturated. Increasing the 440 nm preexcitation intensity $I_{440}$, with fixed $I_{520}$, results in a negligible increase in the RYDMR amplitude beyond $I_{440}\;\sim 1\;{W/cm}^{2}$.

The value of $B_{0}$ at which the resonance feature occurs was investigated by testing samples in RF resonators with different center frequencies. The measured values of the line-center field $B_{0res}$ at different RF frequencies are plotted in Fig. 3 A, showing good agreement with the theoretical prediction for the ESR. Furthermore, if the static field is parallel to the RF field, we do not observe any obvious resonance features (Fig. 3 B) as expected from the ESR transition selection rules.

We have observed MFEs and RYDMR from multiple RFPs together with FMN. The largest MFEs are measured for mScarlet and its variants (mScarlet-I, mScarlet3) at the ~ 20 % level (Fig. 2 and SI) and the smallest for mCherry at ~ 1.5 %. Similarly, the largest RYDMR features are measured in mScarlet and its variants reaching almost 10% increase in fluorescence (Fig. 2 A, B). Measurements for mCherry, and two of it variants show that mutations can have an effect on MFE and RYDMR characteristics (see SI).

Our setup enables detection of magnetic resonance dependent fluorescence in the widefield, as the BLGR geometry inherently provides a near-uniform oscillating magnetic field amplitude $B_{1}$ over the area enclosed by the resonator (25), and the Helmholtz coils provide $B_{0}$ uniformity better than 0.3 % over a volume of ~ (1.5 cm)^3^ (see SI). These features enable measurement of RYDMR from multiple *C. elegans* nematodes simultaneously (Fig. 4). Spatially-resolved RYDMR measurements are obtained by measuring the fluorescence from a specified region of interest of the image, while the magnetic field $B_{0}$ is varied. Mapping out the RYDMR amplitude over the entire image (Fig. 4 C and F) shows that measurement of RYDMR is spatially correlated with mScarlet fluorescence originating from the nematodes. In comparison to the 20 % level MFEs measured in vitro (Fig. 2A and D), the largest MFEs measured in *C. elegans* are around 4 %. One possible explanation might be the lower levels of endogenous free flavin cofactor in the animals. However, the magnetic field at which the RYDMR resonance occurs, $B_{0res}$, is largely insensitive to the cofactor concentration.

We have also investigated the engineered flavin-binding fluorescent protein MagLOV (27) and an mScarlet-MagLOV fusion construct which possibly show reduced cofactor concentration dependence of the MFE and RYDMR amplitude as the flavin is collocated with the RFP (Fig. 1 B). Measurements of RYDMR from *E. coli* colonies expressing these engineered proteins are given in the SI.

The characteristics of the magnetic resonance features in the RFP-flavin system suggest that a spin-correlated radical pair is responsible for the observed magnetic effects. A proposed simplified reaction scheme is illustrated in Fig. S3. Following initial photoexcitation of the FMN cofactor near its absorption peak (∼ 440 nm), subsequent photoexcitation with green light results in the formation of a SCRP. This is likely due to electron transfer or hydrogen atom abstraction with an amino acid(s) on the surface of the RFP by the excited flavin or its photoproduct, as shown in early photochemically-induced dynamic nuclear polarization (photoCIDNP) studies of amino acids:flavin systems (29, 30). Note that the physics underlying CIDNP is the same RP mechanism that underlies the magnetic field effects. At low external magnetic fields, hyperfine coupling to magnetic nuclei in the radical partners drives coherent singlet (S) to triplet (T) interconversion of the two-electron spin state of the radical pair (Fig. 1 D). Singlet RPs are returned to their molecular ground states through a reverse reaction. Triplet RPs can return to the ground state either via conversion to S through coherent spin-state mixing or through incoherent spin relaxation on much slower time scales. In this model, we assume that the ground state of the RFP is the only form of the RFP that can be excited to a state that fluoresces appreciably upon illumination with green light. The resulting fluorescence is sensitive to the spin state of the radical pair, as it affects the population of the ground state of the RFP. This could occur by direct involvement of the RFP chromophore or indirectly by interaction of the fluorescing state of RFP with one partner of the RP, e.g. a free radical on the surface of the RFP. In the absence of an applied magnetic field, the RP triplet sublevels $T_{-1},\;$ $T_{0},\;$ and $T_{1}$ are close to degeneracy and coherent interconversion can occur between S and all triplet sublevels (Fig. 1 D Case i). If an external magnetic field with flux density $B_{0}$ is applied, the energy splitting between the triplet sublevels increases due to the Zeeman effect. If $B_{0}$ is increased such that Zeeman splitting exceeds the hyperfine coupling driving the coherent spin-state mixing, the $T_{\pm 1}$ levels become energetically isolated and coherent interconversion can only occur appreciably between the S and $T_{0}$ states (Fig. 1 D Case ii). Thus, for a radical pair born in the triplet state, an external magnetic field will reduce the steady state population of ground-state RFPs resulting in a reduction of measurable fluorescence (Fig 1 D Case ii). Note that there is a smooth decrease in fluorescence as $B_{0}$ increases, so there is no evidence for residual exchange coupling in the RP. If an RF magnetic field $B\left(t\right)=B_{1}\;sin(f_{RF}t)$, is applied in a direction orthogonal to $B_{0}$, where the frequency $f_{RF}$ matches the electron Larmor frequency, transitions between $T_{0}$ and $T_{\pm 1}$ can occur. The coupling between triplet states by the RF field enables population in the $T_{\pm 1}$ states to access $T_{0}$ and consequently S (Fig 1 D, case iii), resulting in an increase in fluorescence when the RF field frequency is near the electron Larmor frequency (Fig 1 D). The resonance fluorescence linewidth can be used to give an estimate of the coherence lifetime of the radical pair. The narrowest measured FWHM of 2.7(7) mT (Fig. 2 C) corresponds to a frequency linewidth of Δv=72MHz and a coherence time of $1/\pi \Delta \nu \;=\;4\left(1\right)ns$ — potentially enabling radical-pair-based biosensing applications (31).

Our work also suggests that RFPs could serve as genetically-encoded fluorophores for various magnetic imaging modalities that have traditionally relied on chemical agents unsuitable for biological applications (32). FP mutants with enhanced magnetic resonance characteristics and field sensitivity could be generated using directed evolution approaches (27).

We have demonstrated that RF magnetic fields can modulate reaction dynamics in a protein system at room temperature in vitro and in a living animal. This opens up opportunities for designing RF-controllable genetically-encoded systems for regulation of other biomolecular processes such as cell signaling or gene expression. We anticipate that engineering genetically-encoded quantum entangled states in biological systems could enable interfacing living organisms with emerging quantum technologies.

## Supplementary Material

Supplement 1

## Figures and Tables

**Figure 1:**
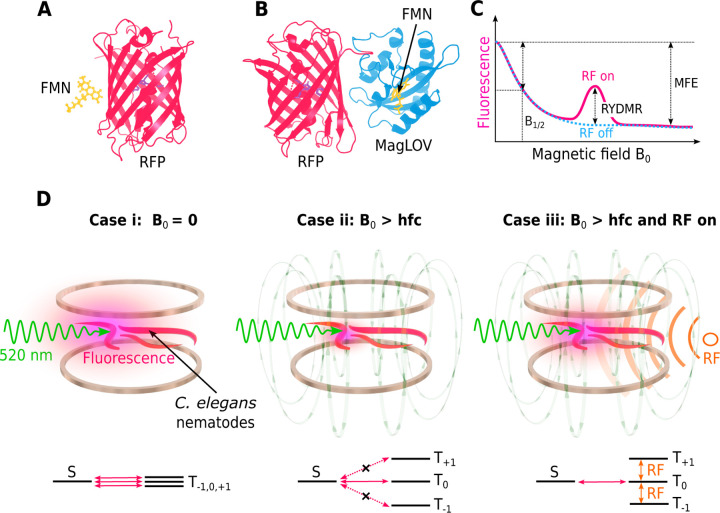
Modulation of fluorescence from red fluorescent proteins (RFPs) in a fluorescent protein: flavin system using radio-frequency magnetic fields. (**A**) Structure of mScarlet (PDB ID: 5LK4) together with an illustration of flavin mononucleotide (FMN). (**B**) Rendering of mScarlet-MagLOV fusion protein (PDB IDs: 5LK4 and 7PGY). (**C**) Schematic of predicted RFP fluorescence intensity as a function of the static magnetic field strength $B_{0}$, with continuous RF applied at the ESR frequency. $B_{1/2}$ is the magnetic field at which the fluorescence has decreased by half of the maximum magnetic field effect (MFE). The increase in the fluorescence resulting from the RF is the reaction yield detected magnetic resonance (RYDMR). (**D**) Illustrations of *C. elegans* nematodes expressing RFP fluorescing under green light excitation are shown in the upper row. Pre-excitation with blue light (not shown) is used for flavin photoproduct generation from endogenous flavin prior to green light excitation. Helmholtz coils generate a magnetic field $B_{0}$. Corresponding singlet $\left(S\right)$ and triplet (T) energy levels from a putative spin-correlated radical-pair are shown beneath. With $B_{0}=0,\;$ S-T mixing can occur between S and all triplet sublevels (Case i). When $B_{0}$ exceeds the nuclear hyperfine constants (hfc) that drive S-T mixing (Case ii), the $T_{\pm 1}$ sublevels become energetically isolated and only $T_{0}$ can be converted to S efficiently, resulting in a reduction of RFP fluorescence. Application of an RF magnetic field at the ESR frequency redistributes population between the triplet sublevels, resulting in increased fluorescence (Case iii).

**Figure 2:**
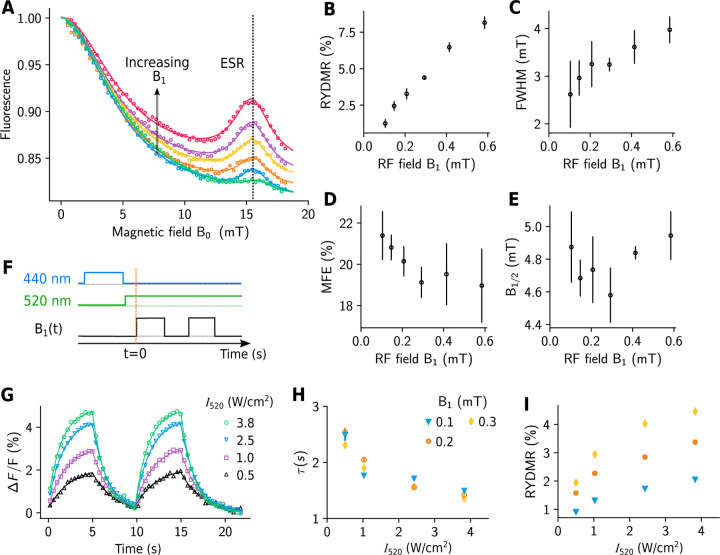
RYDMR from an aqueous solution of purified mScarlet-I and FMN. (**A**) Fractional changes in mScarlet-I fluorescence at various values of the DC field $B_{0⟘}$ in the presence of a 447 MHz RF field with increasing values of $B_{1}$ ranging from 0.07 to 0.37 mT. Solid lines are fits to the data as described in the main text and SI. (**B**) to (**E**), Resonance amplitude (RYDMR), full width at half maximum (FWHM), magnetic field effect (MFE), and $B_{1/2}$ derived from the fit parameters (see Fig. 1 C and SI). Error bars indicate the estimated standard errors from three technically repeated experiments. The vertical dashed line in **A** indicates the ESR magnetic field (15.9 mT) corresponding to 447 MHz. (**F**) Timing diagram (not to scale) for measuring changes in fluorescence resulting from step changes in the RF field (between a specified value of $B_{1}$ and 0 mT) with $B_{0⟘}=15.9\;mT$ at various 520 nm intensities $\left(I_{520}\right)$. (**G**) Time traces showing fractional changes in fluorescence (Δ*F*/*F*) with $B_{1}=0.3\;mT$ from t = 0 – 5 s and 10 – 15 s and $B_{1}=0$ elsewhere. Solid lines are fits to experimental data (see SI) to estimate resonance fluorescence time constants (**H**) and amplitudes (**I**) at various values of $I_{520}$ and $B_{1}$. For all experiments, concentrations of mScarlet-I and FMN are 50 *μ*M and 350 *μ*M respectively. Experiments were conducted at 21.5° C.

**Figure 3:**
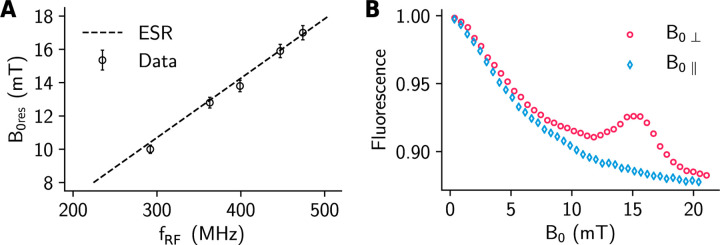
Electron spin resonance. (**A**) Measured resonance magnetic field $B_{0res}$ at various values of the RF drive frequency. The dashed line is the ESR magnetic field strength as a function of the RF frequency calculated from ${B_{0}=h\;f}_{RF/}\mu_{0}g$. Error bars indicate the uncertainty in the magnetic field calibration. (**B**) Measured fluorescence at various values of the static field, parallel $\left(B_{0ǁ}\right)$ or perpendicular $\left(B_{0⟘}\right)$ to the RF field $B_{1}$.

**Figure 4:**
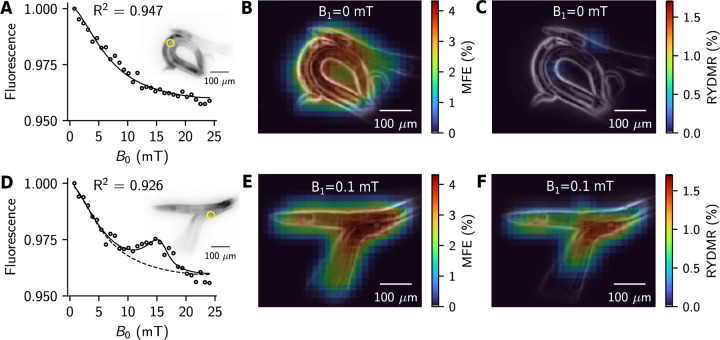
Magnetic field effects and RYDMR in *C. elegans* expressing mScarlet in all cells. (**A**) to (**C**) Experimental data where the RF is off, $B_{1}=0\;mT$. (**D**) to (**F**) $B_{1}=0.1\;mT$ at 445 MHz. (**A**) and (**D**) Fluorescence from *C. elegans* at various values of the static magnetic field $B_{0}$. The insets show grayscale mScarlet fluorescence images of the nematodes used for the experiments. Fluorescence from the regions within the yellow circles in the insets is plotted in the respective main panels. The solid line is a fit to the data (black circles) as described in the SI. The dashed line indicates the estimated fluorescence with no RF. (**B**) and (**E**) MFE distribution overlaid on an edge map (EM) derived from the fluorescence images of the nematodes shown in **A** and **D**. (**C**) and (**F**) RYDMR distribution overlaid on the EM of the nematodes.
