# Supplementary material for: Magnetic resonance control of reaction yields through genetically-encoded protein:flavin spin-correlated radicals in a live animal
Source: bioRxiv. 2025 Mar 3:2025.02.27.640669. Preprint. [Version 1] doi: 10.1101/2025.02.27.640669 (PMC11908193; doi:10.1101/2025.02.27.640669)
Supplement: Supplement 1 [file NIHPP2025.02.27.640669v1-supplement-1.pdf]

bioRxiv preprint doi: https://doi.org/10.1101/2025.02.27.640669; this version posted March 3, 2025. The copyright holder for this preprint (which was not certified by peer review) is the author/funder, who has granted bioRxiv a license to display the preprint in perpetuity. It is made available under aCC-BY 4.0 International license.

Supplementary Materials for

Magnetic resonance control of reaction yields through genetically-encoded protein:flavin spin-correlated radicals in a live animal

Shaun C. Burd*†, Nahal Bagheri†, Maria Ingaramo, Alec F. Condon, Samsuzzoha Mondal, Dara P. Dowlatshahi, Jacob A. Summers, Srijit Mukherjee, Andrew G. York, Soichi Wakatsuki, Steven G. Boxer, Mark Kasevich

*Corresponding author. Email: scburd@stanford.edu

†These authors contributed equally to this work.

This PDF file includes:

Materials and Methods

Figures S1 to S10

bioRxiv preprint doi: https://doi.org/10.1101/2025.02.27.640669; this version posted March 3, 2025. The copyright holder for this preprint (which was not certified by peer review) is the author/funder, who has granted bioRxiv a license to display the preprint in perpetuity. It is made available under aCC-BY 4.0 International license.

Supplementary Materials

Contents

1 Materials and Methods **S3**

1.1 Experimental setup S3

1.2 RF electronics S4

1.3 Data analysis and fitting procedures S4

1.4 Red Fluorescent Proteins S6

1.4.1 Materials, plasmids and protein sequences S6

1.4.2 Protein expression and purification S12

1.4.3 MFEs and RYDMR in other RFPs S13

1.5 Proposed mechanism for MFEs in RFP-flavin systems S13

1.5.1 Absorption measurements S15

1.5.2 Fluorescence measurements S16

1.6 Caenorhabditis elegans S17

1.6.1 Preparation of transgenic *C. elegans* expressing mScarlet in all cells S17

1.6.2 *C. elegans* RYDMR experiments S17

1.6.3 Wild-type *C. elegans* autofluorescence S18

1.7 MagLOV and mScarlet-MagLOV fusion proteins S18

1.7.1 Plasmids, sequences and *E. coli* colony preparation S18

1.7.2 MFEs and RYDMR S20

2 Supplementary Figures **S21**

bioRxiv preprint doi: https://doi.org/10.1101/2025.02.27.640669; this version posted March 3, 2025. The copyright holder for this preprint (which was not certified by peer review) is the author/funder, who has granted bioRxiv a license to display the preprint in perpetuity. It is made available under aCC-BY 4.0 International license.

1 Materials and Methods

1.1 Experimental setup

The experimental setup is illustrated in Fig. S1. Static magnetic fields along the x ($B_{0\,\|}$) and z ($B_{0\,\perp}$) directions are generated using pairs of coils in the Helmholtz configuration. Programmable power supplies (Keysight E36233A and E36155A) enable generation of fields ranging from 0 to 25 mT along x and 0 to 30 mT along z. Calibration of the magnetic field and measurements of magnetic field spatial uniformity are performed using a Texas Instruments TMAG5273A1 3-axis Hall-effect sensor.

Oscillating magnetic fields along the x direction are generated using a bridged loop-gap resonator (BLGR). The resonator used for the results in Fig. 2 is constructed from a 32 mm length copper tube with an inner (outer) diameter of 31 mm (37 mm). A single $\sim$ 0.6 mm wide gap is cut along the tube's length and filled with polytetrafluoroethylene (PTFE) dielectric. A curved copper bridge, 10 mm wide and 30 mm long, is positioned symmetrically over the gap on the outside of the tube, with PTFE dielectric separating the bridge from the tube. Resonators with modified geometries were used to achieve different resonance frequencies for the data shown in Fig. 3 **A**. We note that the BLGR is very effective at heating the air within the RF shield. To prevent undesirable sample heating, the RF shield is continuously flushed with filtered dry air.

Fiber-coupled multimode laser diodes at 440 nm (Wavespectrum RLS/445NM-3500MW) and 520 nm (Wavespectrum RLS/520NM-800MW) generate excitation light. We note that an additional 561 nm source (Coherent Sapphire) was used for *C. elegans* experiments where mentioned. The lasers are combined using a multimode fiber combiner (Thorlabs MP3LF1) to ensure coaxial propagation of all wavelengths onto the sample. A dichroic beamsplitter (Semrock FF593-Di03-25x36 for experiments with RFPs and MagLOV-mScarlet fusion or Thorlabs DMLP505 for MagLOV) reflects the laser light and transmits epi-fluorescence. For experiments involving purified RFPs and *E. coli* colonies, fluorescence is collected using a zoom lens (Thorlabs MVL7002) and imaged onto a FLIR BFS-U3-32S4M-C camera. For experiments with purified RFPs in Figs. 2 **G - I** and for *C. elegans* experiments, fluorescence is collected using a 0.1 NA stereo microscope objective lens placed between the sample and the dichroic. Images are recorded using a pco.edge 5.5 sCMOS camera.

bioRxiv preprint doi: https://doi.org/10.1101/2025.02.27.640669; this version posted March 3, 2025. The copyright holder for this preprint (which was not certified by peer review) is the author/funder, who has granted bioRxiv a license to display the preprint in perpetuity. It is made available under aCC-BY 4.0 International license.

1.2 RF electronics

RF signals are initially generated using an HP E4421B synthesizer. The synthesizer output is amplified using a Mini-Circuits ZHL-100W-GaN+ amplifier. For transient experiments an RF switch (Mini-Circuits ZYSW-2-50DR) placed before the amplifier enables 5 ns switching of the RF using TTL logic signals. Back reflections of RF to the amplifier are limited by placing an RF circulator after the amplifier. After the circulator, a dual directional coupler (HP 778D) samples both the forward and reflected power from the resonator (using Mini-Circuits ZX47-50LN+ power detectors). These measurements were used to calculate the power dissipated in the resonator. The direct output port of the coupler is connected to a coaxial cable terminated in a $\sim$ 25 mm inner diameter coupling loop positioned below the BLGR. Optimization of the coupling is achieved by varying the distance between the loop and the resonator.

The quality factor (Q) of the BLGR resonator was determined by measuring the duration τ for the RF voltage amplitude across the resonator coupling loop to reduce by a factor of $1/e$ after switching off the RF. The quality factor can then be calculated using $Q = 2\pi f_{\mathrm{RF}}\tau/2$. The RF magnetic field amplitude at the sample position B_1 is related to the dissipated power (P) by $B_1 = \eta\sqrt{P}$. Using the method of perturbing spheres (*33, 34*), we measure $\eta = 0.075\,\mathrm{mT}/\sqrt{\mathrm{W}}$.

1.3 Data analysis and fitting procedures

Experimental data showing B_0 dependent fluorescence are recorded while a sequence of sawtooth current ramps are applied to the Helmholtz coils to generate a time-dependent magnetic field profile $B_0(t)$.

Effects of laser-induced fluorophore bleaching are partially removed by subtracting a function $\mathcal{F}_b(B_0(t))$. For in vitro experiments this function is a straight line fitted between data points where $B_0 = 0$ at either ends of each current ramp sequence. For experiments with *C. elegans* and for transient-response experiments (Figs. **G - I**), a function consisting of the sum of an exponential decay and a third-order polynomial is used to characterize the bleaching for two consecutive current ramps sequences.

After subtraction of the bleaching function data sets are analyzed by nonlinear least squares fitting (Scipy.optimize.curve_fit) using the function

bioRxiv preprint doi: https://doi.org/10.1101/2025.02.27.640669; this version posted March 3, 2025. The copyright holder for this preprint (which was not certified by peer review) is the author/funder, who has granted bioRxiv a license to display the preprint in perpetuity. It is made available under aCC-BY 4.0 International license.

$$f(B_0, a, b, c, d, e) = a\left(\mathcal{L}_0(B_0, b) - \mathcal{L}_0(0, b)\right) + c\left(\mathcal{L}_1(0, d, e) - \mathcal{L}_1(B_0, d, e)\right) \tag{S1}$$

where

$$\mathcal{L}_0(B_0, b) = \frac{1}{1 + \left(\dfrac{B_0}{b}\right)^2} \quad \text{and} \quad \mathcal{L}_1(B_0, d, e) = \frac{1}{1 + \left(\dfrac{B_0 - d}{e}\right)^2}$$

are Lorentzian functions that empirically model the MFE and the magnetic resonance respectively. The fitting algorithm returns a set of optimal fit parameters $\mathbf{p}^* = \{a^*, b^*, c^*, d^*, e^*\}$ and a covariance matrix **cov**. Properties of the MFE and the resonance (Figs 2 **B**-**E** and 3**A**) are related to the fit parameters by $B_{1/2} = b^*$, $B_{0\text{res}} = d^*$, and FWHM $= 2e^*$. The MFE is the fraction change in fluorescence due to the magnetic field

$$\text{MFE} = \frac{a*}{\mathcal{F}_b(B_0)}, \tag{S2}$$

and the RYDMR amplitude is given by

$$\text{RYDMR} = \frac{c*}{\mathcal{F}_b(B_{0\text{res}}) - a^*}. \tag{S3}$$

where the denominator is the estimate of the fluorescence with only the static field. Standard errors for the fit parameters are obtained from the square root of the diagonal elements of the covariance matrix $\boldsymbol{\sigma}^* = \{\sigma_a, \sigma_b, \sigma_c, \sigma_d\, \sigma_e\} = \sqrt{diag(\mathbf{cov})}$. Uncertainties associated with parameters derived from the optimal fit parameters are calculated using error propagation formulae (*35*).

The fitted values of the MFE in Fig. 2 **D** are extrapolations as B_0 cannot be increased beyond about 30 mT in our setup. However, the fitted values of the MFE shown in Fig. 2 **D** are consistent with measurements of the MFE performed using a ~ 100 mT magnetic field with a solution of mScarlet-I and FMN (see S 1.5.2).

Transient-response experiments demonstrate that there is a delayed response in fluorescence intensity to a step change of the RF field (Fig. 2 **G**). This effect was incorporated in the fitting procedure by applying a first-order, low-pass filter with time constant τ to Eq. S1 before each evaluation of the objective function in the fitting algorithm. The values of τ obtained from the fit

bioRxiv preprint doi: https://doi.org/10.1101/2025.02.27.640669; this version posted March 3, 2025. The copyright holder for this preprint (which was not certified by peer review) is the author/funder, who has granted bioRxiv a license to display the preprint in perpetuity. It is made available under aCC-BY 4.0 International license.

are shown in Fig. 2 **H**. The low-pass filtering effect results in a slight reduction in the measured value of $B_{0\text{res}}$ ($\sim 2\,\%$ for $\tau \sim 1.5$ s) and in the measured RYDMR amplitude. Scan periods for $B_0(t)$ were selected to be ~ 1 min - significantly longer than the $\tau = 1.2$ s required for the fluorescence to reach as new steady state after a step change in the RF.

1.4 Red Fluorescent Proteins

1.4.1 Materials, plasmids and protein sequences

Flavin Mononucleotide (FMN) was purchased from Aaron Chemicals (catalog number: AR00AEZ8).

All RFP plasmids were based on the pBAD backbone, except for mScarlet3, which was constructed on the pET-28a(+) plasmid. To avoid repetition, the complete plasmid sequences for mScarlet and mScarlet3 are detailed below, while only the protein sequences are provided for all other RFPs used in this study.

Whole plasmid sequence for mScarlet in pBAD backbone:

```
gccgacatcaccgatggggaagatcgggctcgccacttcgggctcatgagcgcttgtttcggcgtgggtatggtggcagg
ccccgtggccgggggactgttgggcgccatctccttctgcctcgcgcgtttcggtgatgacggtgaaaacctctgacaca
tgcagctcccggagacggtcacagcttgtctgtaagcggatgccgggagcagacaagcccgtcagggcgcgtcagcgggt
gttggcgggtgtcggggcgcagccatgacccagtcacgtagcgatagcggagtgtatactggcttaactatgcggcatca
gagcagattgtactgagagtgcaccagatgcggtgtgaaataccgcacagatgcgtaaggagaaaataccgcatcaggcg
ctcttccgcttcctcgctcactgactcgctgcgctcggtcgttcggctgcggcgagcggtatcagctcactcaaaggcgg
taatacggttatccacagaatcaggggataacgcaggaaagaacatgtgagcaaaaggccagcaaaaggccaggaaccgt
aaaaaggccgcgttgctggcgtttttccataggctccgcccccctgacgagcatcacaaaaatcgacgctcaagtcagag
gtggcgaaacccgacaggactataaagataccaggcgtttccccctggaagctccctcgtgcgctctcctgttccgaccc
tgccgcttaccggatacctgtccgcctttctcccttcgggaagcgtggcgctttctcatagctcacgctgtaggtatctc
agttcggtgtaggtcgttcgctccaagctgggctgtgtgcacgaaccccccgttcagcccgaccgctgcgccttatccgg
taactatcgtcttgagtccaacccggtaagacacgacttatcgccactggcagcagccactggtaacaggattagcagag
cgaggtatgtaggcggtgctacagagttcttgaagtggtggcctaactacggctacactagaaggacagtatttggtatc
tgcgctctgctgaagccagttaccttcggaaaaagagttggtagctcttgatccggcaaacaaaccaccgctggtagcgg
tggtttttttgtttgcaagcagcagattacgcgcagaaaaaaaggatctcaagaagatcctttgatcttttctacggggt
ctgacgctcagtggaacgaaaactcacgttaagggattttggtcatgagattatcaaaaaggatcttcacctagatcctt
```

bioRxiv preprint doi: https://doi.org/10.1101/2025.02.27.640669; this version posted March 3, 2025. The copyright holder for this preprint (which was not certified by peer review) is the author/funder, who has granted bioRxiv a license to display the preprint in perpetuity. It is made available under aCC-BY 4.0 International license.

ttaaattgtaaacgttaatattttgttaaaattcgcgttaaatttttgttaaatcagctcattttttaaccaataggccg

aaatcggcaaaatcccttataaatcaaaagaatagcccgagatagggttgagtgttgttccagtttggaacaagagtcca

ctattaaagaacgtggactccaacgtcaaagggcgaaaaaccgtctatcagggcgatggcccactacgtgaaccatcacc

caaatcaagttttttggggtcgaggtgccgtaaagcactaaatcggaaccctaaagggagcccccgatttagagcttgac

ggggaaagccggcgaacgtggcgagaaaggaagggaagaaagcgaaaggagcgggcgctagggcgctggcaagtgtagcg

gtcacgctgcgcgtaaccaccacacccgccgcgcttaatgcgccgctacagggcgcgtaaatcaatctaaagtatatatg

agtaaacttggtctgacagttaccaatgcttaatcagtgaggcacctatctcagcgatctgtctatttcgttcatccata

gttgcctgactccccgtcgtgtagataactacgatacgggagggcttaccatctggccccagtgctgcaatgataccgcg

agacccacgctcaccggctccagatttatcagcaataaaccagccagccggaagggccgagcgcagaagtggtcctgcaa

ctttatccgcctccatccagtctattaattgttgccgggaagctagagtaagtagttcgccagttaatagtttgcgcaac

gttgttgccattgctgcaggcatcgtggtgtcacgctcgtcgtttggtatggcttcattcagctccggttcccaacgatc

aaggcgagttacatgatcccccatgttgtgcaaaaaagcggttagctccttcggtcctccgatcgttgtcagaagtaagt

tggccgcagtgttatcactcatggttatggcagcactgcataattctcttactgtcatgccatccgtaagatgcttttct

gtgactggtgagtactcaaccaagtcattctgagaatagtgtatgcggcgaccgagttgctcttgcccggcgtcaacacg

ggataataccgcgccacatagcagaactttaaaagtgctcatcattggaaaacgttcttcggggcgaaaactctcaagga

tcttaccgctgttgagatccagttcgatgtaacccactcgtgcacccaactgatcttcagcatcttttactttcaccagc

gtttctgggtgagcaaaaacaggaaggcaaaatgccgcaaaaaagggaataagggcgacacggaaatgttgaatactcat

actcttcctttttcaatattattgaagcatttatcagggttattgtctcatgagcggatacatatttgaatgtatttaga

aaaataaacaaaagagtttgtagaaacgcaaaaaggccatccgtcaggatggccttctgcttaatttgatgcctggcagt

ttatggcgggcgtcctgcccgccaccctccgggccgttgcttcgcaacgttcaaatccgctcccggcggatttgtcctac

tcaggagagcgttcaccgacaaacaacagataaaacgaaaggcccagtctttcgactgagcctttcgttttatttgatgc

ctggcagttccctactctcgcatggggagaccccacactaccatcggcgctacggcgtttcacttctgagttcggcatgg

ggtcaggtgggaccaccgcgctactgccgccaggcaaattctgttttatcagaccgcttctgcgttctgatttaatctgt

atcaggctgaaaatcttctctcatccgccaaaacagccaagcttcgaattcttacttgtacagctcgtccatgccgccgg

tggagtggcggccctcggagcgttcgtactgttccaccacggtgtagtcctcgttgtgggaggtgatgtccaacttgcgg

tcgacgttgtaggcgccgggcatctgcacgggcttcttggccttgtaggtggtcttgaagtccgccaggtagcggccgcc

gtccttcaggcgcagggccatcttaatgtcgcccttcagcacgccgtcctcggggtacaaccgctcggtggacgcttccc

agcccattgtcttcttctgcattacggggccgtcaggagggaagttggtgccgcggagcttcaccttgtagatcagggtg

ccgtcctccagggaggtgtcctgggtcacggtcacggcgccgccgtcctcgaagttcatcacgcgctcccacttgaagcc

ctcggggaaggactgcttatagtagtcggggatgtcggcggggtgcttggtgaaggccctggagccgtacatgaactgag

bioRxiv preprint doi: https://doi.org/10.1101/2025.02.27.640669; this version posted March 3, 2025. The copyright holder for this preprint (which was not certified by peer review) is the author/funder, who has granted bioRxiv a license to display the preprint in perpetuity. It is made available under aCC-BY 4.0 International license.

gggacaggatgtcccaggagaagggcagggggccacccttggtcaccttcagcttggcggtctgggtgccctcgtagggg
cggccctcgccctcgccctcgatctcgaactcgtggccgttcatggagccctccatgtgcaccttgaaccgcatgaactc
cttgatcactgcctcgcccttgctcaccatttttttgggatccttatcgtcatcgtcgtacagatcccgacccatttgct
gtccaccagtcatgctagccataccatgatgatgatgatgatgagaaccccgcatatgtatatctccttcttaaagttaa
acaaaattatttctagcccaaaaaaacgggtatggagaaacagtagagagttgcgataaaaagcgtcaggtagtatccgc
taatcttatggataaaaatgctatggcatagcaaagtgtgacgccgtgcaaataatcaatgtggacttttctgccgtgat
tatagacacttttgttacgcgtttttgtcatggctttggtcccgctttgttacagaatgcttttaataagcggggttacc
ggtttggttagcgagaagagccagtaaaagacgcagtgacggcaatgtctgatgcaatatggacaattggtttcttctct
gaatggcgggagtatgaaaagtatggctgaagcgcaaaatgatcccctgctgccgggatactcgtttaatgcccatctgg
tggcgggtttaacgccgattgaggccaacggttatctcgatttttttatcgaccgaccgctgggaatgaaaggttatatt
ctcaatctcaccattcgcggtcaggggtggtgaaaaatcagggacgagaatttgtttgccgaccgggtgatattttgct
gttcccgccaggagagattcatcactacggtcgtcatccggaggctcgcgaatggtatcaccagtgggtttactttcgtc
cgcgcgcctactggcatgaatggcttaactggccgtcaatatttgccaatacggggttctttcgcccggatgaagcgcac
cagccgcatttcagcgacctgtttgggcaaatcattaacgccgggcaaggggaagggcgctattcggagctgctggcgat
aaatctgcttgagcaattgttactgcggcgcatggaagcgattaacgagtcgctccatccaccgatggataatcgggtac
gcgaggcttgtcagtacatcagcgatcacctggcagacagcaattttgatatcgccagcgtcgcacagcatgtttgcttg
tcgccgtcgcgtctgtcacatcttttccgccagcagttagggattagcgtcttaagctggcgcgaggaccaacgtatcag
ccaggcgaagctgcttttgagcaccacccggatgcctatcgccaccgtcggtcgcaatgttggttttgacgatcaactct
atttctcgcgggtatttaaaaaatgcaccggggccagcccgagcgagttccgtgccggttgtgaagaaaaagtgaatgat
gtagccgtcaagttgtcataattggtaacgaatcagacaattgacggcttgacggagtagcatagggtttgcagaatccc
tgcttcgtccatttgacaggcacattatgcatcgatgataagctgtcaaacatgagcagatcctctacgccggacgcatc
gtggccggcatcaccggcgccacaggtgcggttgctggcgcctatatc

mScarlet Protein Sequence:

MRGSHHHHHH GMASMTGGQQ MGRDLYDDDD KDPKKMVSKG EAVIKEFMRF KVHMEGSMNG
HEFEIEGEGE GRPYEGTQTA KLKVTKGGPL PFSWDILSPQ FMYGSRAFTK HPADIPDYYK
QSFPEGFKWE RVMNFEDGGA VTVTQDTSLE DGTLIYKVKL RGTNFPPDGP VMQKKTMGWE
ASTERLYPED GVLKGDIKMA LRLKDGGRYL ADFKTTYKAK KPVQMPGAYN VDRKLDITSH
NEDYTVVEQY ERSEGRHSTG GMDELYK

bioRxiv preprint doi: https://doi.org/10.1101/2025.02.27.640669; this version posted March 3, 2025. The copyright holder for this preprint (which was not certified by peer review) is the author/funder, who has granted bioRxiv a license to display the preprint in perpetuity. It is made available under aCC-BY 4.0 International license.

mScarlet-I Protein Sequence:

MRGSHHHHHH GMASMTGGQQ MGRDLYDDDD KDPKKMVSKG EAVIKEFMRF KVHMEGSMNG

HEFEIEGEGE GRPYEGTQTA KLKVTKGGPL PFSWDILSPQ FMYGSRAFIK HPADIPDYYK

QSFPEGFKWE RVMNFEDGGA VTVTQDTSLE DGTLIYKVKL RGTNFPPDGP VMQKKTMGWE

ASTERLYPED GVLKGDIKMA LRLKDGGRYL ADFKTTYKAK KPVQMPGAYN VDRKLDITSH

NEDYTVVEQY ERSEGRHSTG GMDELYK

mCherry Protein Sequence:

MRGSHHHHHH GMASMTGGQQ MGRDLYDDDD KDPMVSKGEE DNMAIIKEFM RFKVHMEGSV

NGHEFEIEGE GEGRPYEGTQ TAKLKVTKGG PLPFAWDILS PQFMYGSKAY VKHPADIPDY

LKLSFPEGFK WERVMNFEDG GVVTVTQDSS LQDGEFIYKV KLRGTNFPSD GPVMQKKTMG

WEASSERMYP EDGALKGEIK QRLKLKDGGH YDAEVKTTYK AKKPVQLPGA YNVNIKLDIT

SHNEDYTIVE QYERAEGRHS TGGMDELYK

mCherry-XL Protein Sequence:

MRGSHHHHHH GMASMTGGQQ MGRDLYDDDD KDPKKMVSKG EEDNMAIIKE FMRFKVHMEG

SVNGHEFEIE GEGEGRPYEG TQTAKLKVTK GGPLPFAWDI LSPQFMYGSK AYVKHPADIP

DYLKLSFPEG FKWERVMNFE DGGVVTVTQD SSLQDGEFIY KVKLKGTNFP SDGPVMQKKT

MGSEASSERM YPEDGALKGE VKYRLKLKDG GHYDAEVKTT YKAKKPVQLP GAYNVNRKLD

ITSHNEDYTI VEQYERAEGR HSTGGMDELY K

mCherry-D Protein Sequence:

MRGSHHHHHH GMASMTGGQQ MGRDLYDDDD KDPKKMVSKA EEDNMAIIKE FMRFKTRMEG

SVNGHEFEIE GEGEGRPYEG TQTAKLKVTK GGPLPFAWDI LSPQFMYGSR AYVKHPADIP

DYLKLSFPEG FKWERVMKSE DGGVVTVTQD SSLQDGEFIY KVKLRGTNFP SDGPVMQKKT

MGWEASSERM YPEDGALKGE MKMRLRLKDG GHYDWEVKTT YKAKKPVQLP GAYNVNRKLD

ITSHNEDYTI VEQYERAEGR HSTGGMDELY K

Whole plasmid sequence for mScarlet3 in pET-28a(+) backbone:

tggcgaatgggacgcgccctgtagcggcgcattaagcgcggcgggtgtggtggttacgcgcagcgtgaccgctacacttg

bioRxiv preprint doi: https://doi.org/10.1101/2025.02.27.640669; this version posted March 3, 2025. The copyright holder for this preprint (which was not certified by peer review) is the author/funder, who has granted bioRxiv a license to display the preprint in perpetuity. It is made available under aCC-BY 4.0 International license.

ccagcgccctagcgcccgctcctttcgctttcttcccttcctttctcgccacgttcgccggctttccccgtcaagctcta
aatcgggggctccctttagggttccgatttagtgctttacggcacctcgaccccaaaaaacttgattagggtgatggttc
acgtagtgggccatcgccctgatagacggtttttcgccctttgacgttggagtccacgttctttaatagtggactcttgt
tccaaactggaacaacactcaaccctatctcggtctattcttttgatttataagggattttgccgatttcggcctattgg
ttaaaaaatgagctgatttaacaaaaatttaacgcgaattttaacaaaatattaacgtttacaatttcaggtggcacttt
tcggggaaatgtgcgcggaacccctatttgtttatttttctaaatacattcaaatatgtatccgctcatgaattaattct
tagaaaaactcatcgagcatcaaatgaaactgcaatttattcatatcaggattatcaataccatatttttgaaaaagccg
tttctgtaatgaaggagaaaactcaccgaggcagttccataggatggcaagatcctggtatcggtctgcgattccgactc
gtccaacatcaatacaacctattaatttcccctcgtcaaaaataaggttatcaagtgagaaatcaccatgagtgacgact
gaatccggtgagaatggcaaaagtttatgcatttctttccagacttgttcaacaggccagccattacgctcgtcatcaaa
atcactcgcatcaaccaaaccgttattcattcgtgattgcgcctgagcgagacgaaatacgcgatcgctgttaaaaggac
aattacaaacaggaatcgaatgcaaccggcgcaggaacactgccagcgcatcaacaatattttcacctgaatcaggatat
tcttctaatacctggaatgctgttttcccggggatcgcagtggtgagtaaccatgcatcatcaggagtacggataaaatg
cttgatggtcggaagaggcataaattccgtcagccagtttagtctgaccatctcatctgtaacatcattggcaacgctac
ctttgccatgtttcagaaacaactctggcgcatcgggcttcccatacaatcgatagattgtcgcacctgattgcccgaca
ttatcgcgagcccatttatacccatataaatcagcatccatgttggaatttaatcgcggcctagagcaagacgtttcccg
ttgaatatggctcataacaccccttgtattactgtttatgtaagcagacagttttattgttcatgaccaaaatcccttaa
cgtgagttttcgttccactgagcgtcagaccccgtagaaaagatcaaaggatcttcttgagatcctttttttctgcgcgt
aatctgctgcttgcaaacaaaaaaaccaccgctaccagcggtggtttgtttgccggatcaagagctaccaactctttttc
cgaaggtaactggcttcagcagagcgcagataccaaatactgtccttctagtgtagccgtagttaggccaccacttcaag
aactctgtagcaccgcctacatacctcgctctgctaatcctgttaccagtggctgctgccagtggcgataagtcgtgtct
taccgggttggactcaagacgatagttaccggataaggcgcagcggtcgggctgaacggggggttcgtgcacacagccca
gcttggagcgaacgacctacaccgaactgagatacctacagcgtgagctatgagaaagcgccacgcttcccgaagggaga
aaggcggacaggtatccggtaagcggcagggtcggaacaggagagcgcacgagggagcttccagggggaaacgcctggta
tctttatagtcctgtcgggtttcgccacctctgacttgagcgtcgatttttgtgatgctcgtcaggggggcggagcctat
ggaaaaacgccagcaacgcggcctttttacggttcctggccttttgctggccttttgctcacatgttctttcctgcgtta
tcccctgattctgtggataaccgtattaccgcctttgagtgagctgataccgctcgccgcagccgaacgaccgagcgcag
cgagtcagtgagcgaggaagcggaagagcgcctgatgcggtattttctccttacgcatctgtgcggtatttcacaccgca
tatatggtgcactctcagtacaatctgctctgatgccgcatagttaagccagtatacactccgctatcgctacgtgactg
ggtcatggctgcgccccgacacccgccaacacccgctgacgcgccctgacgggcttgtctgctcccggcatccgcttaca

bioRxiv preprint doi: https://doi.org/10.1101/2025.02.27.640669; this version posted March 3, 2025. The copyright holder for this preprint (which was not certified by peer review) is the author/funder, who has granted bioRxiv a license to display the preprint in perpetuity. It is made available under aCC-BY 4.0 International license.

gacaagctgtgaccgtctccgggagctgcatgtgtcagaggttttcaccgtcatcaccgaaacgcgcgaggcagctgcgg

taaagctcatcagcgtggtcgtgaagcgattcacagatgtctgcctgttcatccgcgtccagctcgttgagtttctccag

aagcgttaatgtctggcttctgataaagcgggccatgttaagggcggtttttcctgtttggtcactgatgcctccgtgt

aagggggatttctgttcatgggggtaatgataccgatgaaacgagagaggatgctcacgatacgggttactgatgatgaa

catgcccggttactggaacgttgtgagggtaaacaactggcggtatggatgcggcgggaccagagaaaaatcactcaggg

tcaatgccagcgcttcgttaatacagatgtaggtgttccacagggtagccagcagcatcctgcgatgcagatccggaaca

taatggtgcagggcgctgacttccgcgtttccagactttacgaaacacggaaaccgaagaccattcatgttgttgctcag

gtcgcagacgttttgcagcagcagtcgcttcacgttcgctcgcgtatcggtgattcattctgctaaccagtaaggcaacc

ccgccagcctagccgggtcctcaacgacaggagcacgatcatgcgcacccgtggggccgccatgccggcgataatggcct

gcttctcgccgaaacgtttggtggcgggaccagtgacgaaggcttgagcgagggcgtgcaagattccgaataccgcaagc

gacaggccgatcatcgtcgcgctccagcgaaagcggtcctcgccgaaaatgacccagagcgctgccggcacctgtcctac

gagttgcatgataaagaagacagtcataagtgcggcgacgatagtcatgccccgcgcccaccggaaggagctgactgggt

tgaaggctctcaagggcatcggtcgagatcccggtgcctaatgagtgagctaacttacattaattgcgttgcgctcactg

cccgctttccagtcgggaaacctgtcgtgccagctgcattaatgaatcggccaacgcgcggggagaggcggtttgcgtat

tgggcgccagggtggtttttcttttcaccagtgagacgggcaacagctgattgcccttcaccgcctggccctgagagagt

tgcagcaagcggtccacgctggtttgccccagcaggcgaaaatcctgtttgatggtggttaacggcgggatataacatga

gctgtcttcggtatcgtcgtatcccactaccgagatatccgcaccaacgcgcagcccggactcggtaatggcgcgcattg

cgcccagcgccatctgatcgttggcaaccagcatcgcagtgggaacgatgccctcattcagcatttgcatggtttgttga

aaaccggacatggcactccagtcgccttcccgttccgctatcggctgaatttgattgcgagtgagatatttatgccagcc

agccagacgcagacgcgccgagacagaacttaatgggcccgctaacagcgcgatttgctggtgacccaatgcgaccagat

gctccacgcccagtcgcgtaccgtcttcatgggagaaaataatactgttgatgggtgtctggtcagagacatcaagaaat

aacgccggaacattagtgcaggcagcttccacagcaatggcatcctggtcatccagcggatagttaatgatcagcccact

gacgcgttgcgcgagaagattgtgcaccgccgctttacaggcttcgacgccgcttcgttctaccatcgacaccaccacgc

tggcacccagttgatcggcgcgagatttaatcgccgcgacaatttgcgacggcgcgtgcagggccagactggaggtggca

acgccaatcagcaacgactgtttgcccgccagttgttgtgccacgcggttgggaatgtaattcagctccgccatcgccgc

ttccactttttcccgcgttttcgcagaaacgtggctggcctggttcaccacgcgggaaacggtctgataagagacaccgg

catactctgcgacatcgtataacgttactggtttcacattcaccaccctgaattgactctcttccgggcgctatcatgcc

ataccgcgaaaggttttgcgccattcgatggtgtccgggatctcgacgctctcccttatgcgactcctgcattaggaagc

agcccagtagtaggttgaggccgttgagcaccgccgccgcaaggaatggtgcatgcaaggagatggcgcccaacagtccc

ccggccacggggcctgccaccatacccacgccgaaacaagcgctcatgagcccgaagtggcgagcccgatcttccccatc

bioRxiv preprint doi: https://doi.org/10.1101/2025.02.27.640669; this version posted March 3, 2025. The copyright holder for this preprint (which was not certified by peer review) is the author/funder, who has granted bioRxiv a license to display the preprint in perpetuity. It is made available under aCC-BY 4.0 International license.

```
ggtgatgtcggcgatataggcgccagcaaccgcacctgtggcgccggtgatgccggccacgatgcgtccggcgtagagga
tcgagatctcgatcccgcgaaattaatacgactcactataggggaattgtgagcggataacaattcccctctagaaataa
ttttgtttaactttaagaaggagatataccatgaggggatcacatcaccaccatcaccactctagcggtttggttccgcg
tgcggttattaaagagttcatgcgttttaaggtgcacatggaagggtctatgaatggtcatgaattcgagatcgaaggcg
agggcgagggtcgtccgtacgaaggcacccagaccgcgaagctgcgcgttaccaaaggtggtccgctgccgtttagctgg
gatattctgtccccgcaatttatgtatggtagccgtgccttcaccaagcacccggcggacatcccggactactggaaaca
atcgttcccggaaggtttcaagtgggagcgcgtgatgaactttgaggacggcggcgcggtgagcgttgcgcaggatacct
ccttggaagacggcactctgatttataaagtcaaattgcgcggtacgaacttccctccggatggcccagtaatgcagaaa
aagacgatgggttgggaggctagcaccgaacgtttatatccggaggacgtcgttctgaagggtgatatcaaaatggcact
gagacttaaggacggcggtcgctacctggcagattttaagaccacgtaccgcgctaaaaagccggtgcagatgccgggtg
cgttcaacattgatcgtaaactggatatcacctcccacaatgaagactacaccgttgtggaacaatatgagcgtagcgtg
gcccgtcatagctaacaaagcccgaaaggaagctgagttggctgctgccaccgctgagcaataactagcataaccccttg
gggcctctaaacgggtcttgaggggttttttgctgaaaggaggaactatatccggat
```

mScarlet3 Protein Sequence:

```
MRGSHHHHHH SSGLVPRAVI KEFMRFKVHM EGSMNGHEFE IEGEGEGRPY EGTQTAKLRV
TKGGPLPFSW DILSPQFMYG SRAFTKHPAD IPDYWKQSFP EGFKWERVMN FEDGGAVSVA
QDTSLEDGTL IYKVKLRGTN FPPDGPVMQK KTMGWEASTE RLYPEDVVLK GDIKMALRLK
DGGRYLADFK TTYRAKKPVQ MPGAFNIDRK LDITSHNEDY TVVEQYERSV ARHS
```

1.4.2 Protein expression and purification

Each RFP plasmid was transformed into *Escherichia coli* BL21(DE3) cells (Invitrogen). Selected colonies were inoculated into two 5 mL cultures of Luria-Bertani (LB) medium supplemented with 100 µg/mL ampicillin and incubated overnight at 37 °C with shaking at 225 rpm. The following day, the overnight cultures were combined and transferred into 1 L of LB medium, supplemented with ampicillin 100 µg/ml. The culture was grown at 37 °C and 220 rpm until the optical density at 600 nm (OD600) reached 0.6. At this point, arabinose was added to a final concentration of 2 mg/ml to induce protein expression. The cells were then incubated overnight at 18 °C with shaking at 220 rpm.

On the next day, the cells were harvested by centrifugation at 4000 rpm for 30 minutes and

bioRxiv preprint doi: https://doi.org/10.1101/2025.02.27.640669; this version posted March 3, 2025. The copyright holder for this preprint (which was not certified by peer review) is the author/funder, who has granted bioRxiv a license to display the preprint in perpetuity. It is made available under aCC-BY 4.0 International license.

stored at -80 °C. For lysis, cells were resuspended in lysis buffer (0.1 M Tris, pH 8.0) and disrupted using a homogenizer set to a pressure of 20 MPa with a flow rate of 35 mL/min. Five passes through the homogenizer were used to ensure complete cell lysis. The lysate was centrifuged at 16,000 rpm for 45 minutes to pellet cellular debris. The supernatant was filtered through a 0.22 µm membrane (Millipore Corp.) and loaded onto a His-tag affinity column pre-equilibrated with lysis buffer. Proteins without His-tag were removed by washing the column with 5 column volumes of wash buffer (0.1 M Tris, 20 mM imidazole, pH 8.0). The target protein was eluted using 3 column volumes of elution buffer (0.1 M Tris, 300 mM imidazole, pH 8.0). Finally, the protein was buffer-exchanged into 0.1 M Tris buffer, pH 8.0.

1.4.3 MFEs and RYDMR in other RFPs

We have measured the MFEs from the emission of various RFPs together with FMN. We note that the magnitude of MFE is influenced by several factors including the mutations of the fluorescent protein, the FMN to protein concentration ratio, temperature, and green and blue optical excitation intensities. Among the RFPs tested, the largest MFEs (measured in the regime where Δg mechanism is insignificant) have been observed in mScarlet and its variants mScarlet-I, mScarlet3, reaching approximately 20 %, while mCherry exhibits the lowest MFE saturating at around 1.5%. MFEs and RYDMR data for mCherry and two variants: mCherry-XL, and mCherry-D (*36, 37*) are shown in Fig. S2. The mutations in these mCherry variants resulted in increases in both the MFE and the RYDMR amplitude compared to mCherry, indicating that mutations can influence the magnetic response of fluorescent proteins. No obvious essential amino acids leading to these differences have been identified to date. To avoid RF heating, all in vitro RYDMR experiments are conducted by dissolving RFP and FMN in distilled water.

1.5 Proposed mechanism for MFEs in RFP-flavin systems

A hypothesized spin-correlated radical pair (RP)-based reaction scheme in the FMN-RFP system is illustrated in Fig. S3. In the investigated RFP-flavin systems, photosensitization with blue light, presumably to convert FMN to an FMN photoproduct, seems to be necessary to observe MFEs. While RFPs can weakly absorb blue light and fluoresce, the largest MFEs in their emission are

bioRxiv preprint doi: https://doi.org/10.1101/2025.02.27.640669; this version posted March 3, 2025. The copyright holder for this preprint (which was not certified by peer review) is the author/funder, who has granted bioRxiv a license to display the preprint in perpetuity. It is made available under aCC-BY 4.0 International license.

observed using green light excitation, following or together with blue light excitation. This suggests RP formation between the RFP and FMN photoproduct during the green light excitation period. Furthermore, after the system has been pre-excited using blue light, introducing a period with no optical excitation ranging from seconds (Fig. S6) to several minutes before green light excitation, still results in MFEs in fluorescence. This suggests that the FMN photoproduct is likely not an FMN excited state. The precise chemical identity of the photoproduct remains unknown, and we tentatively call it flavinX.

Application of green light to a mixture of RFP and flavinX can result in excitation of the RFP chromophore, which we denote RFP^c, to form RFP^{c*} which can fluoresce and return to the RFP^c ground state. We distinguish the protein barrel, denoted RFP, from the RFP chromophore, RFP^c, in the following. Additionally, we hypothesize that green light excitation can lead to the formation of a RP between flavinX, initially in the triplet state ^{3}flavinX, and RFP, as suggested by CIDNP studies of amino acid:flavin systems, where flavin or its photoproduct is formed in the triplet state (*29, 30*). This process likely involves electron transfer or hydrogen atom abstraction from an amino acid on the surface of the RFP by ^{3}flavinX. We invoke the triplet state of flavinX as this is a bimolecular reaction and the likely lifetime of the singlet state of flavinX is too short for RP formation to be efficient. We note that it is possible that the initial spin state of the radical pair is a singlet state, but the same RP would be involved and MFEs would still be expected.

Hyperfine interactions drive coherent interconversion between the singlet state (S) and triplet state (T) of the RP, which can be sensitive to external magnetic fields. From S, the RP can rapidly decay —on nanosecond to microsecond timescales—resulting in repopulation of the RFP ground state. From T, the RP can either convert to S or decay, resulting in a dark species whose chemical identity remains unknown. We hypothesize that this dark species could transition back to the RFP ground state over longer timescales (presumably on the millisecond to second timescales), as ∼10s of cycles of magnetically-induced fluorescence modulation over a total period of several minutes do not lead to a significant decrease in fluorescence intensity or measured MFE for each cycle. The precise feature(s) of the RFP component of the radical pair is not known at this time. This could be a radical on the surface of the protein that affects the excited state properties of RFP^c or it could directly involve the RFP chromophore, e.g. by secondary electron transfer.

While still preliminary, aspects of this model are supported by in vitro absorption and fluores-

bioRxiv preprint doi: https://doi.org/10.1101/2025.02.27.640669; this version posted March 3, 2025. The copyright holder for this preprint (which was not certified by peer review) is the author/funder, who has granted bioRxiv a license to display the preprint in perpetuity. It is made available under aCC-BY 4.0 International license.

cence measurements performed on solutions of RFP with FMN. These measurements are described in detail in the subsections below and the key observations are summarized here. During the blue light excitation period, fluorescence measurements (Fig. S6 B) show a reduction in the concentration of ground state oxidized FMN (fluorescence maximum near 530 nm), indicating the formation of a photoproduct. With the blue light source switched off, applying green light results in an initial decrease in mScarlet fluorescence (Fig. S6 B) and a decrease in absorption (Fig. S5 A) associated with the fluorescent protein (around 569 nm). This reduction in absorption is inversely correlated with the formation of a new absorption peak centered near 520 nm (Fig. S5 A). When an external magnetic field is applied, RFP fluorescence and absorption (around 569 nm) both decrease (up to ~20 %), while the absorption feature at 520 nm increases. We hypothesize that the 520 nm feature corresponds to the dark species mentioned above. Spectrally resolved fluorescence measurements show no obvious spectral shifts in the mScarlet emission resulting from the magnetic field (Fig. S6 C). These observations suggest that following FMN photoproduct formation, the magnetic field serves to control the population of RFP molecules that can be excited and fluoresce in the presence of green light.

1.5.1 Absorption measurements

Steady-state absorption spectra (obtained using a Perkin Elmer LAMBDA 365+ UV/Vis Spectrometer) for a mixture of mScarlet and FMN, as well as for the individual components (FMN and mScarlet), are shown in Fig. S4. indicating no significant alteration in their absorption properties due to potential interactions.

Time-resolved absorption spectra of the mScarlet and FMN mixture are presented in Fig. S5. Spectra were recorded following 30 s pre-excitation with a 440 nm laser with intensity at the sample of 9 W/cm^2. Subsequently, probe light from a 3500 K white LED was used to irradiate the sample. Simultaneously with the probe, light from a 520 nm laser with intensity 7 W/cm^2 was incident on the sample. This source was pulsed with a frequency of 10 Hz and a 50 % duty cycle. Absorption of the white light probe was recorded only during the intervals where the 520 nm laser was off using an OceanFX spectrometer (Ocean Optics). A magnetic field, generated using Helmholtz coils, was switched between 0 and $\sim$ 40 mT with 50 % duty cycle and a 40 s period to enable measurement of magnetic field effects throughout the experiment.

bioRxiv preprint doi: https://doi.org/10.1101/2025.02.27.640669; this version posted March 3, 2025. The copyright holder for this preprint (which was not certified by peer review) is the author/funder, who has granted bioRxiv a license to display the preprint in perpetuity. It is made available under aCC-BY 4.0 International license.

Immediately following the initialization of 520 nm light exposure, there is an absorption maximum at 569 nm, corresponding to the mScarlet absorption peak (Fig.S5 A). As the duration of 520 nm exposure increases, the absorption peak at 569 nm falls almost 50 % after 0.7 s and a new absorption peak is formed near 520 nm (Fig.S5 A). The increase in absorption at 520 nm is commensurate with the decrease in absorption at 569 nm suggesting the formation of a non-fluorescent version of the FP, inversely correlated with the RFP ground state population measured at 569 nm. Furthermore, the change in absorption at 520 and 569 nm induced by the magnetic field are also anticorrelated (Fig.S5 B, C, and D) suggesting that the reduction in RFP fluorescence due to the magnetic field results from a reduction in the population of ground state RFPs that can be excited and fluoresce. Significantly weaker modulation correlated with the magnetic field switching is present at 445 nm (corresponding to the FMN absorption maximum) and at 594 nm (the mScarlet emission maximum).

Control experiments with solutions containing only FMN or mScarlet using the same pre-excitation and green light irradiation showed no evidence of increased absorption at 520 nm. To rule out potential artifacts from the 520 nm excitation laser, the experiments were repeated using a 569 nm laser instead. The longer wavelength excitation source results in no significant changes to the results shown in Fig. S5 or to their interpretation.

1.5.2 Fluorescence measurements

Spectrally-resolved fluorescence from a solution of 50 μM mScarlet-I and 300 μM FMN was measured during a period of blue light excitation and subsequently during a period of green light excitation (Fig. S6). A magnetic field is switched between 0 and ~100 mT every 90 s throughout the experiment by periodically moving a permanent magnet close to the sample. A 450 nm light emitting diode (LED), with an 18 nm FWHM bandwidth and a 530 nm LED with a 35 nm FWHM bandwidth were used as excitation sources. Fluorescence was measured using an Ocean SR2 spectrometer (Ocean Optics). Rapid quenching of the mScarlet-I fluorescence to ~ 10 % of its initial value occurs within seconds of the start of the excitation period (Fig. S6 B). For the remaining ~ 10 min of blue light exposure, the fluorescence remains suppressed and weak modulation anti-correlated with the switching of the magnetic field is visible. After a period with no excitation, applying green light results in increased fluorescence and an increased magnetic field effect (~20 % reduction in

bioRxiv preprint doi: https://doi.org/10.1101/2025.02.27.640669; this version posted March 3, 2025. The copyright holder for this preprint (which was not certified by peer review) is the author/funder, who has granted bioRxiv a license to display the preprint in perpetuity. It is made available under aCC-BY 4.0 International license.

fluorescence). Representative spectra with and without the magnetic field are given in Fig. S6 C. The magnetic field results in no obvious spectral shifts in the emission spectra.

1.6 Caenorhabditis elegans

1.6.1 Preparation of transgenic *C. elegans* expressing mScarlet in all cells

The worm strain WBM1143 [eft-3p::3XFLAG::wrmScarlet::unc-54 3'UTR *wbmIs65] (*24*) was used for the ubiquitous expression of a worm codon optimized version of mScarlet. This strain was obtained from the from Caenorhabditis Genetics Center (CGC). *C. elegans* were grown and maintained at 20 °C on nematode growth media (NGM) plates supplemented with the OP50 strain of *E. coli* as the food source (*38*). For imaging, larval stage 4 (L4) and day one adults were mounted in 4% agarose and paralyzed with levamisole (1 mM).

1.6.2 *C. elegans* RYDMR experiments

MFEs and RYDMR were measured in multiple *C. elegans* samples with $B_1 = 0.1$ mT (Fig. S7) and $B_1 = 0$ mT (Fig. S8). To increase fluorescence intensity and signal-to-noise ratio, in addition to the 520 nm laser used in previous measurements, we employed a 561 nm laser for green light excitation, with a total green laser intensity of ~ 40 W/cm^2. For each sample, fluorescence images were recorded at various values of B_0. Fitting Eq. S1 with first-order low-pass filtering to the integrated fluorescence from a region of interest (ROI) in the image gives an estimate of the MFE and the RYDMR for that ROI (Fig. S7, Columns 1 and 2). Maps of the MFE and RYDMR are obtained by dividing the image into blocks and estimating the MFE and RYDMR in each one (Fig. S7, Columns 3 and 4). Figs. 4, S7, and S8 only show RYDMR estimates from blocks where the coefficient of determination $R^2 > 0.6$ and $p_F < 0.01$. The p_{F} value is measured by performing an F test (*39*) to determine whether the addition of fit parameters modeling the RYDMR (c, d and e in Eq. S1) significantly improve the fit. This is done to exclude blocks where the fit performs poorly such as in regions with low fluorescence away from the nematodes. The RYDMR and MFE distributions shown in Fig. 4 and Fig. S9 are smoothed with a 1 sigma Gaussian filter (scipy.ndimage.gaussian_filter). The unfiltered RYDMR distribution corresponding to Fig. 4 F is shown in Fig. S7 row 1 column 4.

bioRxiv preprint doi: https://doi.org/10.1101/2025.02.27.640669; this version posted March 3, 2025. The copyright holder for this preprint (which was not certified by peer review) is the author/funder, who has granted bioRxiv a license to display the preprint in perpetuity. It is made available under aCC-BY 4.0 International license.

1.6.3 Wild-type *C. elegans* autofluorescence

Experiments with wild-type (WT) *C. elegans* were performed to determine if the autofluorescence from WT nematodes exhibited any magnetic-field sensitivity as has been observed in mammalian cells (*40*). No significant MFEs or RYDMR in the autofluorescence of WT worms were observed using the previously described experimental and analysis procedures for the mScarlet expressing nematodes. We performed additional measurements where the usual 650 nm LP fluorescence filter was replaced with a 550 nm LP filter to collect more autofluorescence and improve the measurement signal-to-noise ratio. In these measurements, 561 nm excitation was not used. We measure changes in fluorescence consistent with MFEs of up to 0.6 % in isolated locations in WT samples (Fig. S9 A). In comparison, up to 4 % MFEs were measured using the same 550 nm LP filter with an mScarlet-expressing sample, with a similar spatial distribution to measurements obtained using the 650 nm filter. These experiments clearly demonstrate that the MFEs and RYDMR measured in mScarlet-expressing *C. elegans* is largely due to the presence of the RFP.

1.7 MagLOV and mScarlet-MagLOV fusion proteins

1.7.1 Plasmids, sequences and *E. coli* colony preparation

Both MagLOV and mScarlet-MagLOV constructs are based on the pRSET plasmids. To avoid repetition, the full plasmid sequence for MagLOV is provided, while only the protein sequence for mScarlet-MagLOV is detailed below.

Whole Plasmid Sequence for MagLOV in pRSET Backbone:

```
GATCTCGATCCCGCGAAATTAATACGACTCACTATAGGGAGACCACAACGGTTTCCCTCTAGAAATAATTTTGTTTAACT
TTAAGAAGGAGATATACATATGCGGGGTTCTCATCATCATCATCATCATGGTATGGCTAGCATGCTTGCTACTACTTTAG
AACGTATAGAGAAAAACTTCGTGATCACGGACCCGAGACTACCTGACAACCCTATAATTTTTGCAAGTGACTCATTCCTT
CAGTTGACTGAGTATTCTAGGGAAGAGATTCTAGGGtggAATcCTAGATTCTTGCAAGGACCAGAAACTGACCGTGCCAC
TGTGAGGAAAATCAGGGATGCGATCGACAACCAAACCGAGGTGACAGTGCAGCTAATAAATTACACTAAATCTGGCAAGA
AGTTCTGGAACCTATTTCATgTGCAACCCATGAGAGACCAAAAAGGAGACGTACAGTACTTCATAGGGGTAaagTTGGAT
GGTACTGAGCATGTTAGAGACGCGGCAGAACGTGAAcGGGTTATGTTAATAAAAAAGACCGCTGAAAACATAatgGAAGC
GGCAAAGGAGTTGTAACTCGAGATCTGCAGCTGGTACCATGGAATTCGAAGCTTGATCCGGCTGCTAACAAAGCCCGAAA
```

bioRxiv preprint doi: https://doi.org/10.1101/2025.02.27.640669; this version posted March 3, 2025. The copyright holder for this preprint (which was not certified by peer review) is the author/funder, who has granted bioRxiv a license to display the preprint in perpetuity. It is made available under aCC-BY 4.0 International license.

GGAAGCTGAGTTGGCTGCTGCCACCGCTGAGCAATAACTAGCATAACCCCTTGGGGCCTCTAAACGGGTCTTGAGGGGTT
TTTTGCTGAAAGGAGGAACTATATCCGGATCTGGCGTAATAGCGAAGAGGCCCGCACCGATCGCCCTTCCCAACAGTTGC
GCAGCCTGAATGGCGAATGGGACGCGCCCTGTAGCGGCGCATTAAGCGCGGCGGGTGTGGTGGTTACGCGCAGCGTGACC
GCTACACTTGCCAGCGCCCTAGCGCCCGCTCCTTTCGCTTTCTTCCCTTCCTTTCTCGCCACGTTCGCCGGCTTTCCCCG
TCAAGCTCTAAATCGGGGGCTCCCTTTAGGGTTCCGATTTAGTGCTTTACGGCACCTCGACCCCAAAAAACTTGATTAGG
GTGATGGTTCACGTAGTGGGCCATCGCCCTGATAGACGGTTTTTCGCCCTTTGACGTTGGAGTCCACGTTCTTTAATAGT
GGACTCTTGTTCCAAACTGGAACAACACTCAACCCTATCTCGGTCTATTCTTTTGATTTATAAGGGATTTTGCCGATTTC
GGCCTATTGGTTAAAAAATGAGCTGATTTAACAAAAATTTAACGCGAATTTTAACAAAATATTAACGCTTACAATTTAGG
TGGCACTTTTCGGGGAAATGTGCGCGGAACCCCTATTTGTTTATTTTTCTAAATACATTCAAATATGTATCCGCTCATGA
GACAATAACCCTGATAAATGCTTCAATAATATTGAAAAAGGAAGAGTATGAGTATTCAACATTTCCGTGTCGCCCTTATT
CCCTTTTTTGCGGCATTTTGCCTTCCTGTTTTTGCTCACCCAGAAACGCTGGTGAAAGTAAAAGATGCTGAAGATCAGTT
GGGTGCACGAGTGGGTTACATCGAACTGGATCTCAACAGCGGTAAGATCCTTGAGAGTTTTCGCCCCGAAGAACGTTTTC
CAATGATGAGCACTTTTAAAGTTCTGCTATGTGGCGCGGTATTATCCCGTATTGACGCCGGGCAAGAGCAACTCGGTCGC
CGCATACACTATTCTCAGAATGACTTGGTTGAGTACTCACCAGTCACAGAAAAGCATCTTACGGATGGCATGACAGTAAG
AGAATTATGCAGTGCTGCCATAACCATGAGTGATAACACTGCGGCCAACTTACTTCTGACAACGATCGGAGGACCGAAGG
AGCTAACCGCTTTTTTGCACAACATGGGGGATCATGTAACTCGCCTTGATCGTTGGGAACCGGAGCTGAATGAAGCCATA
CCAAACGACGAGCGTGACACCACGATGCCTGTAGCAATGGCAACAACGTTGCGCAAACTATTAACTGGCGAACTACTTAC
TCTAGCTTCCCGGCAACAATTAATAGACTGGATGGAGGCGGATAAAGTTGCAGGACCACTTCTGCGCTCGGCCCTTCCGG
CTGGCTGGTTTATTGCTGATAAATCTGGAGCCGGTGAGCGTGGGTCTCGCGGTATCATTGCAGCACTGGGGCCAGATGGT
AAGCCCTCCCGTATCGTAGTTATCTACACGACGGGGAGTCAGGCAACTATGGATGAACGAAATAGACAGATCGCTGAGAT
AGGTGCCTCACTGATTAAGCATTGGTAACTGTCAGACCAAGTTTACTCATATATACTTTAGATTGATTTAAAACTTCATT
TTTAATTTAAAAGGATCTAGGTGAAGATCCTTTTTGATAATCTCATGACCAAAATCCCTTAACGTGAGTTTTCGTTCCAC
TGAGCGTCAGACCCCGTAGAAAAGATCAAAGGATCTTCTTGAGATCCTTTTTTTCTGCGCGTAATCTGCTGCTTGCAAAC
AAAAAAACCACCGCTACCAGCGGTGGTTTGTTTGCCGGATCAAGAGCTACCAACTCTTTTTCCGAAGGTAACTGGCTTCA
GCAGAGCGCAGATACCAAATACTGTTCTTCTAGTGTAGCCGTAGTTAGGCCACCACTTCAAGAACTCTGTAGCACCGCCT
ACATACCTCGCTCTGCTAATCCTGTTACCAGTGGCTGCTGCCAGTGGCGATAAGTCGTGTCTTACCGGGTTGGACTCAAG
ACGATAGTTACCGGATAAGGCGCAGCGGTCGGGCTGAACGGGGGGTTCGTGCACACAGCCCAGCTTGGAGCGAACGACCT
ACACCGAACTGAGATACCTACAGCGTGAGCTATGAGAAAGCGCCACGCTTCCCGAAGGGAGAAAGGCGGACAGGTATCCG
GTAAGCGGCAGGGTCGGAACAGGAGAGCGCACGAGGGAGCTTCCAGGGGGAAACGCCTGGTATCTTTATAGTCCTGTCGG
GTTTCGCCACCTCTGACTTGAGCGTCGATTTTTGTGATGCTCGTCAGGGGGGCGGAGCCTATGGAAAAACGCCAGCAACG

bioRxiv preprint doi: https://doi.org/10.1101/2025.02.27.640669; this version posted March 3, 2025. The copyright holder for this preprint (which was not certified by peer review) is the author/funder, who has granted bioRxiv a license to display the preprint in perpetuity. It is made available under aCC-BY 4.0 International license.

CGGCCTTTTTACGGTTCCTGGCCTTTTGCTGGCCTTTTGCTCACATGTTCTTTCCTGCGTTATCCCCTGATTCTGTGGAT
AACCGTATTACCGCCTTTGAGTGAGCTGATACCGCTCGCCGCAGCCGAACGACCGAGCGCAGCGAGTCAGTGAGCGAGGA
AGCGGAAGAGCGCCCAATACGCAAACCGCCTCTCCCCGCGCGTTGGCCGATTCATTAATGCAG

mScarlet-MagLOV Protein Sequence:

MRGSHHHHHH GMASMVSKGE AVIKEFMRFK VHMEGSMNGH EFEIEGEGEG RPYEGTQTAK
LKVTKGGPLP FSWDILSPQF MYGSRAFTKH PADIPDYYKQ SFPEGFKWER VMNFEDGGAV
TVTQDTSLED GTLIYKVKLR GTNFPPDGPV MQKKTMGWEA STERLYPEDG VLKGDIKMAL
RLKDGGRYLA DFKTTYKAKK PVQMPGAYNV DKKLDITSHN EDYTVVEQYE RSEGRHSTGG
GATGLATTLE RIEKNFVITD PRLPDNPIIF ASDSFLQLTE YSREEILGWN PRFLQGPETD
RATVRKIRDA IDNQTEVTVQ LINYTKSGKK FWNLLHVQPM RDQKGDVQYF IGVKLDGTEH
VRDAAGRERV MLIKKTAENI MEAAKELGTM EFEA

Each plasmid was transformed into BL21(DE3) *E. coli* cells and plated onto LB agarose plates. All MFE and RYDMR measurements were performed after one day of incubation at 37°C and one day of incubation at room temperature on the LB agarose plates.

1.7.2 MFEs and RYDMR

MFE and RYDMR experiments for mScarlet-MagLOV and MagLOV colonied were conducted similarly to the procedures described for RFPs in the main text. For MagLOV samples, only blue excitation light is necessary, and a 500 nm longpass filter is used to capture flavin fluorescence. Representative data from single colonies expressing the mScarlet-MagLOV fusion and MagLOV are shown in Fig. S10 . Our setup allows for the measurement of MFE and RYDMR from multiple *E. coli* colonies simultaneously (Fig. S10), with the potential to be used for directed evolution to identify proteins with optimized magnetic resonance and MFEs. Our results suggest that the static MFEs observed for MagLOV by Ingaramo et al. (*27*) are due to spin-correlated radical pairs.

bioRxiv preprint doi: https://doi.org/10.1101/2025.02.27.640669; this version posted March 3, 2025. The copyright holder for this preprint (which was not certified by peer review) is the author/funder, who has granted bioRxiv a license to display the preprint in perpetuity. It is made available under aCC-BY 4.0 International license.

2 Supplementary Figures

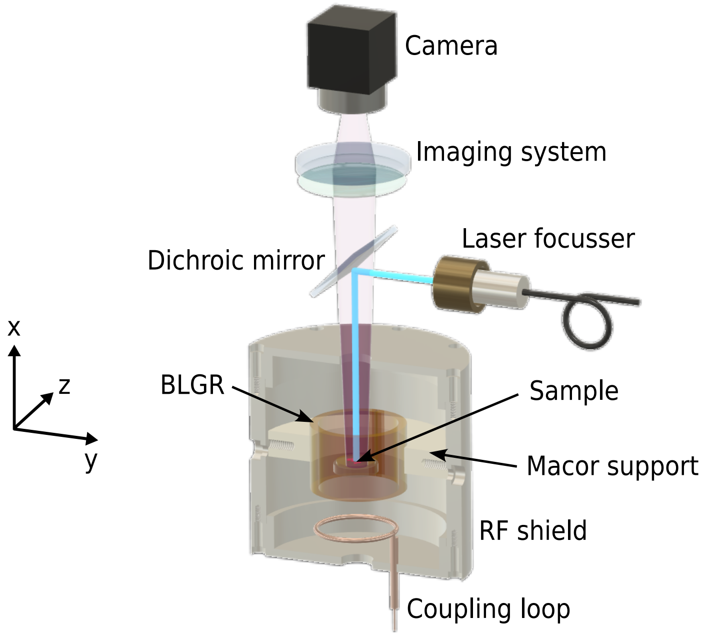

Figure S1: Experimental setup (not to scale). The sample is positioned near the center of a bridged loop-gap resonator (BLGR). Two pairs of Helmholtz coils (not shown) generate static magnetic fields either parallel ($B_{0\,\parallel}$ along x) or perpendicular ($B_{0\,\perp}$ along z) to the RF field direction. A dichroic mirror allows delivery of excitation light at 440, 520, or 561 nm to the sample and transmission of fluorescence to the imaging system and camera.

bioRxiv preprint doi: https://doi.org/10.1101/2025.02.27.640669; this version posted March 3, 2025. The copyright holder for this preprint (which was not certified by peer review) is the author/funder, who has granted bioRxiv a license to display the preprint in perpetuity. It is made available under aCC-BY 4.0 International license.

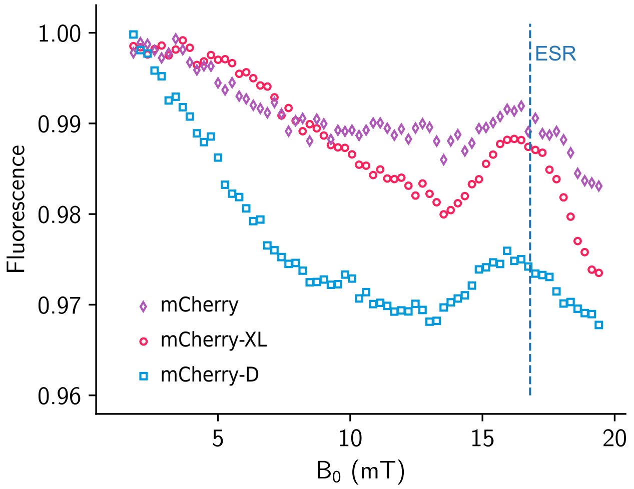

Figure S2: MFEs and RYDMR with mCherry variants. Data points are fluorescence measurements at various values of the static magnetic field B_0 for mCherry (purple diamonds), mCherry-XL (red circles), and mCherry-D (blue squares), in the presence of FMN. The RF field is at 470 MHz, with amplitude $B_1 = 0.1$ mT. The dashed blue line indicates the magnetic field required for ESR at 470 MHz for a $g = 2$ electron spin.

bioRxiv preprint doi: https://doi.org/10.1101/2025.02.27.640669; this version posted March 3, 2025. The copyright holder for this preprint (which was not certified by peer review) is the author/funder, who has granted bioRxiv a license to display the preprint in perpetuity. It is made available under aCC-BY 4.0 International license.

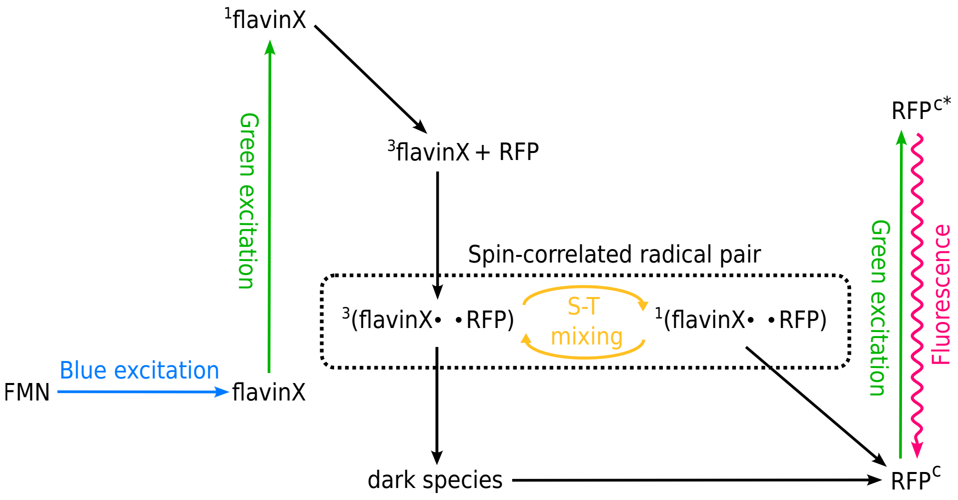

Figure S3: Proposed radical pair reaction scheme for the RFP-flavin system. A two-step process is involved: FMN photoexcitation driven by blue light and a radical pair reaction involving the FMN photoproduct (flavinX) and the RFP. RFP^c is a representative of the RFP chromophore. After photoexcitation with green light, a spin-correlated RP is formed. Hyperfine interactions drive coherent singlet (S) to triplet (T) mixing which is sensitive to magnetic fields. The S state can undergo a rapid reverse reaction returning the RFP to its molecular ground state. From T, the RP can be converted to the S state or decay, resulting in formation of a nonfluorescent version of the RFP (dark species), that can decay back to the RFP ground state on much slower time scales. Application of an external magnetic field results in reduced decay from the S state and a corresponding reduction in the population of ground state RFP molecules. This results in a reduction in fluorescence.

bioRxiv preprint doi: https://doi.org/10.1101/2025.02.27.640669; this version posted March 3, 2025. The copyright holder for this preprint (which was not certified by peer review) is the author/funder, who has granted bioRxiv a license to display the preprint in perpetuity. It is made available under aCC-BY 4.0 International license.

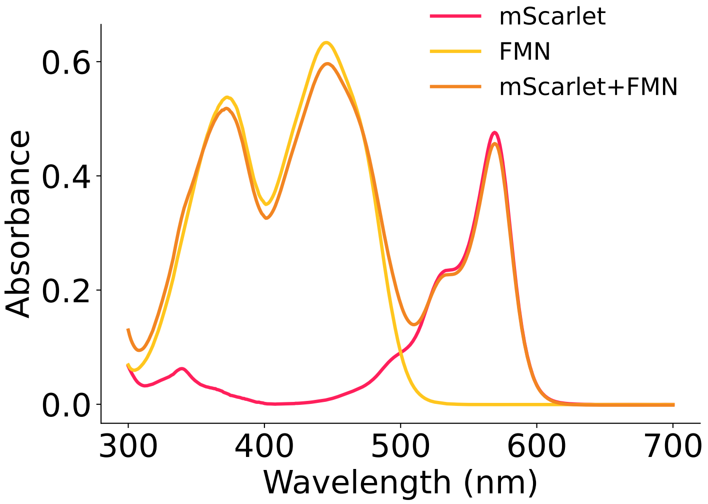

Figure S4: Steady-state absorption spectra for the mixture of mScarlet and FMN, as well as for FMN and mScarlet individually. Concentrations of FMN and mScarlet were about 500 µM and 50 µM, respectively.

bioRxiv preprint doi: https://doi.org/10.1101/2025.02.27.640669; this version posted March 3, 2025. The copyright holder for this preprint (which was not certified by peer review) is the author/funder, who has granted bioRxiv a license to display the preprint in perpetuity. It is made available under aCC-BY 4.0 International license.

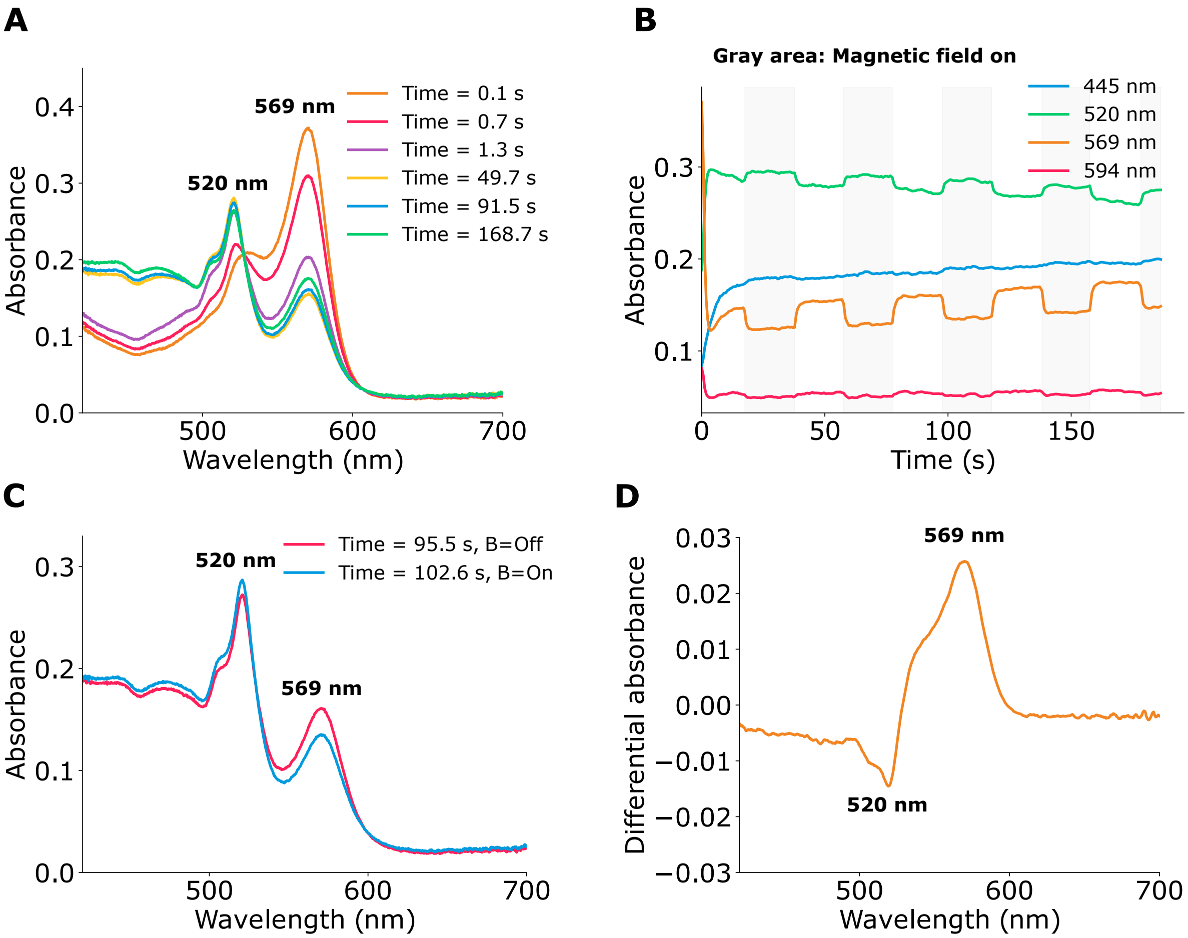

Figure S5: Absorption spectra and magnetically modulated absorption from a solution of mScarlet and FMN. The sample is initially prepared by photoexcitation at 440 nm. (**A**) Absorption from 420 to 700 nm at various durations of 520 nm light exposure. No magnetic field is present in the selected time traces. (**B**) Absorption at various wavelengths as a function of the duration of 520 nm light exposure.White (gray) regions indicate periods when the magnetic field is off (on). (**C**) Absorption with and without the magnetic field. (**D**) Difference in absorption between the traces shown in **C**.

bioRxiv preprint doi: https://doi.org/10.1101/2025.02.27.640669; this version posted March 3, 2025. The copyright holder for this preprint (which was not certified by peer review) is the author/funder, who has granted bioRxiv a license to display the preprint in perpetuity. It is made available under aCC-BY 4.0 International license.

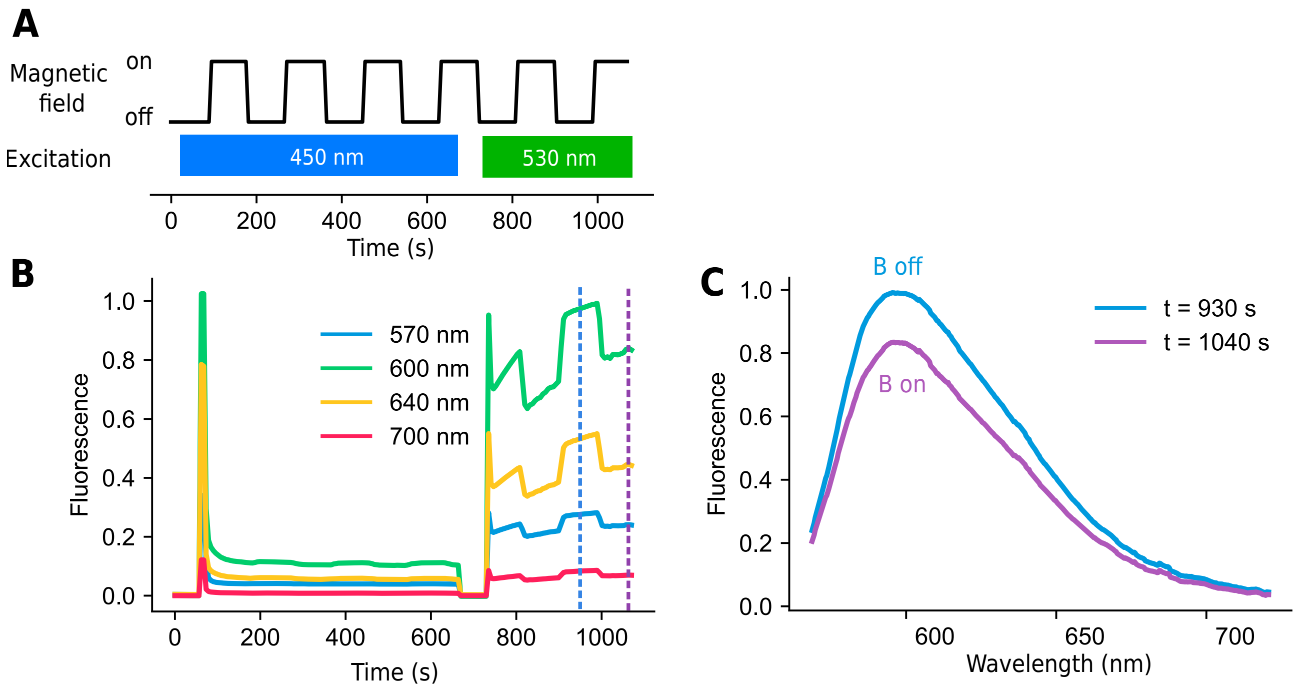

Figure S6: Spectrally-resolved fluorescence magnetic field effects from a solution of 50 µM purified mScarlet-I and 300 µM FMN. (**A**) Timing diagram. The magnetic field is switched between 0 and ~ 100 mT. (**B**) Spectrally-resolved fluorescence as a function of time following the sequence shown in **A**. Emission spectra over the range 570 to 720 nm with the magnetic field off (t = 930 s) and on (t = 1040 s), indicated by dashed vertical lines in **B**.

bioRxiv preprint doi: https://doi.org/10.1101/2025.02.27.640669; this version posted March 3, 2025. The copyright holder for this preprint (which was not certified by peer review) is the author/funder, who has granted bioRxiv a license to display the preprint in perpetuity. It is made available under aCC-BY 4.0 International license.

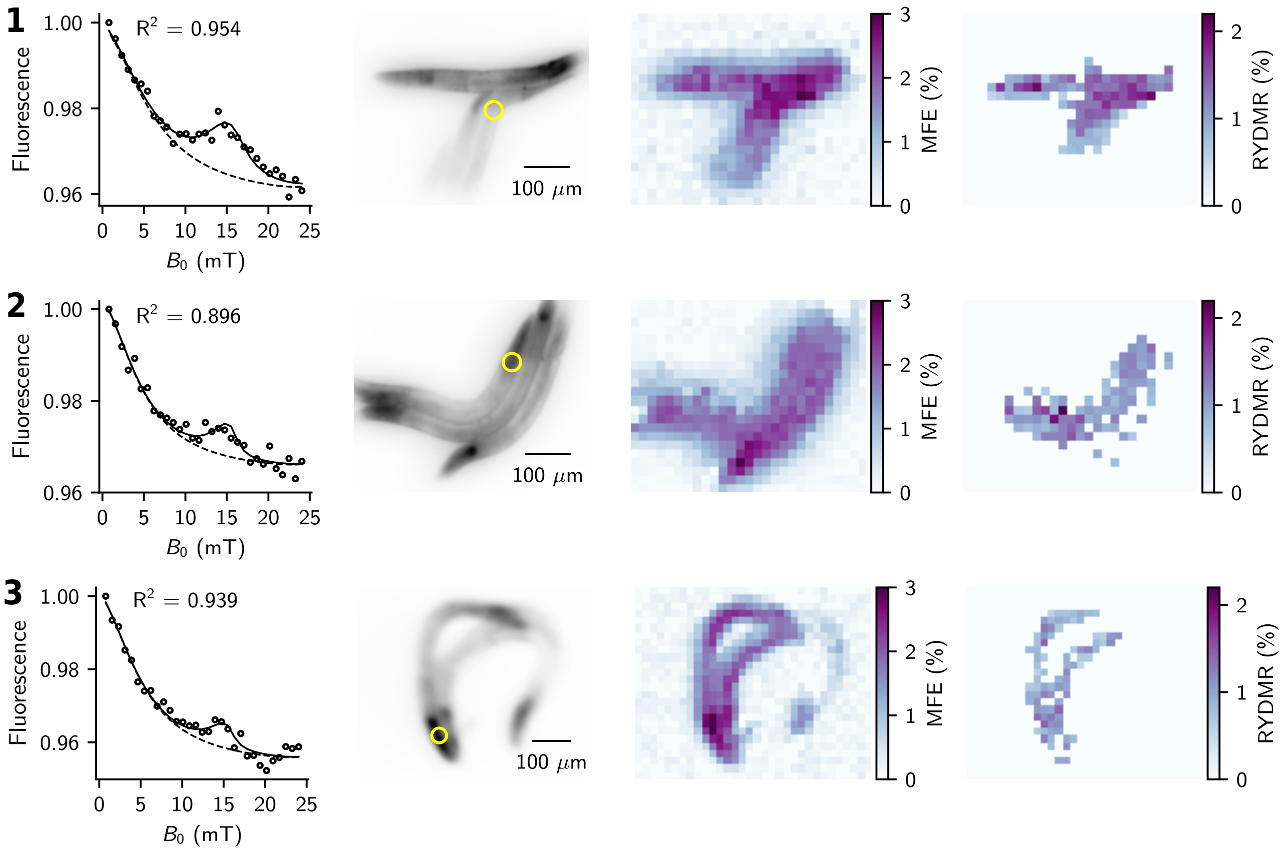

Figure S7: RYDMR from *C. elegans* strain WBM1143. Each row corresponds to a different sample with $B_1 = 0.1$ mT. Column 1, Integrated fluorescence from within the yellow circles shown in the mScarlet fluorescence images in Column 2 at various values of B_0. Data points are given by black circles. Solid black lines are fits of Eq. S1 to the data. Dashed black lines are estimates of the fluorescence with no RF. Column 3, Spatial distribution of the MFE over the image shown in Column 2 obtained by fitting Eq. S1 to the mean fluorescence from $19 \times 19\,\mu$m blocks. Column 4, Spatial distribution of RYDMR amplitudes. Only values where $R^2 > 0.6$ and $p_F < 0.01$ from an F test (see SI text) are shown.

bioRxiv preprint doi: https://doi.org/10.1101/2025.02.27.640669; this version posted March 3, 2025. The copyright holder for this preprint (which was not certified by peer review) is the author/funder, who has granted bioRxiv a license to display the preprint in perpetuity. It is made available under aCC-BY 4.0 International license.

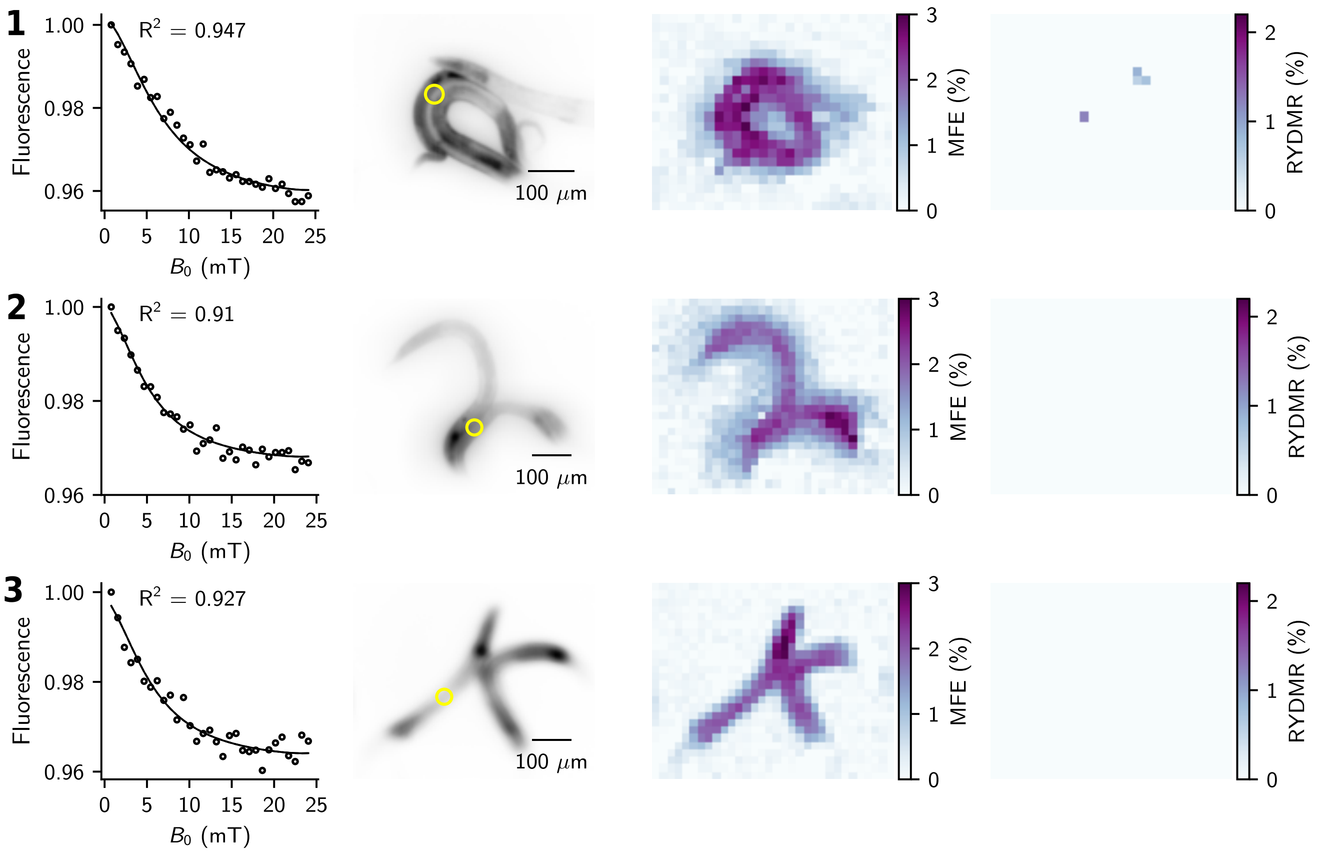

Figure S8: RYDMR from *C. elegans* expressing mScarlet in all cells. Same as for S7, but with $B_1 = 0$

bioRxiv preprint doi: https://doi.org/10.1101/2025.02.27.640669; this version posted March 3, 2025. The copyright holder for this preprint (which was not certified by peer review) is the author/funder, who has granted bioRxiv a license to display the preprint in perpetuity. It is made available under aCC-BY 4.0 International license.

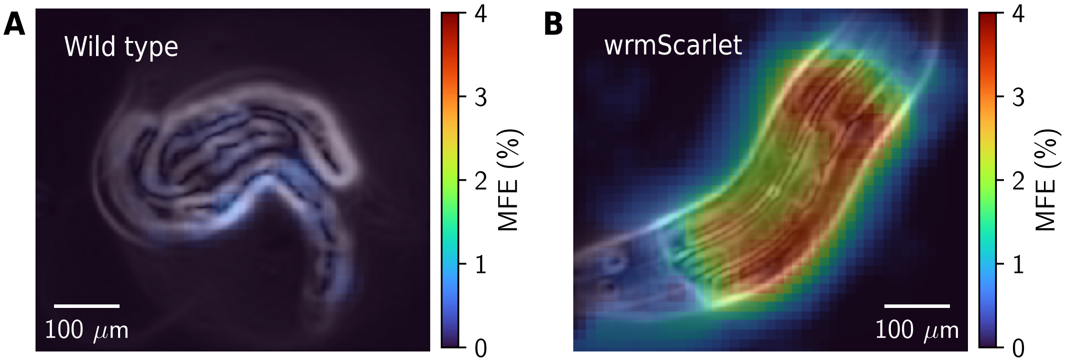

Figure S9: MFEs in fluorescence measured from (**A**) wild-type *C. elegans* and (**B**) *C. elegans* expressing mScarlet in all cells. Each panel shows a color map of the MFE overlaid on an edge map derived from fluorescence images of the nematodes. For these images, fluorescence was collected using a 550 nm long-pass emission filter.

bioRxiv preprint doi: https://doi.org/10.1101/2025.02.27.640669; this version posted March 3, 2025. The copyright holder for this preprint (which was not certified by peer review) is the author/funder, who has granted bioRxiv a license to display the preprint in perpetuity. It is made available under aCC-BY 4.0 International license.

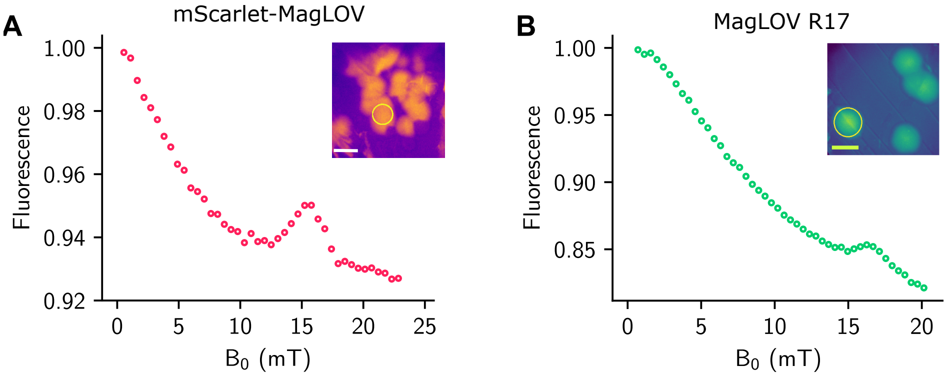

Figure S10: Fluorescence-detected magnetic resonance from *E. coli* colonies expressing mScarlet-MagLOV fusion (**A**) and MagLOV (**B**). Data points are integrated fluorescence from within the regions indicated the yellow circles shown in the insets as B_0 is varied.
